# Ruthenium(II) and Iridium(III) Complexes as Tested Materials for New Anticancer Agents

**DOI:** 10.3390/ma13163491

**Published:** 2020-08-07

**Authors:** Joanna Masternak, Agnieszka Gilewska, Barbara Barszcz, Iwona Łakomska, Katarzyna Kazimierczuk, Jerzy Sitkowski, Joanna Wietrzyk, Anna Kamecka, Magdalena Milczarek

**Affiliations:** 1 Institute of Chemistry, Jan Kochanowski University, Uniwersytecka 7, 25-406 Kielce, Poland; agnieszka5052@wp.pl (A.G.); barbara.barszcz@ujk.edu.pl (B.B.); 2 Faculty of Chemistry, Nicolaus Copernicus University in Toruń, Gagarina 7, 87-100 Toruń, Poland; 3 Department of Inorganic Chemistry, Faculty of Chemistry, Gdańsk University of Technology, Narutowicza 11/12 G., 80-233 Gdańsk, Poland; katarzyna.kazimierczuk@pg.edu.pl; 4 Institute of Organic Chemistry, Polish Academic of Science, Kasprzaka 44/52, 01-224 Warszawa, Poland; j.sitkowski@nil.gov.pl; 5 National Medicines Institute, Chełmska 30/34, 00-725 Warszawa, Poland; 6 Hirszfeld Institute of Immunology and Experimental Therapy, Polish Academy of Sciences, R. Weigla 12, 53-114 Wrocław, Poland; joanna.wietrzyk@hirszfeld.pl (J.W.), magdalena.milczarek@hirszfeld.pl (M.M.); 7 Institute of Chemistry, Faculty of Sciences, University of Natural Sciences and Humanities in Siedlce, 3 Maja 54, 08-110 Siedlce, Poland; anna.kamecka@uph.edu.pl

**Keywords:** iridium(III) complexes, ruthenium half-sandwich, cytotoxicity, DNA interactions

## Abstract

The oncological use of cisplatin is hindered by its severe side effects and a very important resistance problem. To overcome these problems, scientists have attempted to design new generation transition-metal anticancer complexes. In this study, we present new complexes, ruthenium(II) [(η^6^-*p*-cymene)RuCl(py_2_CO)]PF_6_ (**1**), iridium(III) [(η^5^-Cp)IrCl(py_2_CO)]PF_6_ (**2**), and NH_4_[IrCl_4_(py_2_CO)]·H_2_O (**3**), based on di-2-pyridylketone (py_2_CO). The prepared complexes were characterized by FTIR, ^1^H, ^13^C, ^15^N NMR, UV-Vis, PL and elemental analysis techniques. The single-crystal X-ray structure analysis and comparative data revealed pseudo-octahedral half-sandwich **1** and **2** complexes and octahedral tetrachloroiridate(III) **3** with a rare chelating κ^2^N,O coordination mode of py_2_CO. The compounds were tested in vitro against three cancer cell lines—colorectal adenoma (LoVo), myelomonocytic leukaemia (MV-4-11), breast adenocarcinoma (MCF-7), and normal fibroblasts (BALB/3T3). The most promising results were obtained for iridium(III) complex **3** against MV-4-11 (IC_50_ = 35.8 ± 13.9 µg/mL) without a toxic effect against normal BALB/3T3, which pointed towards its selectivity as a potential anticancer agent. Extensive research into their mode of binding with DNA confirmed for **1** and **2** complexes non-classical binding modes, while the 3D circular dichroism (CD) experiment (ΔT_m_) suggested that **3** induced the probable formation of covalent bonds with DNA. In addition, the obtained iridium complexes induce ROS, which, in synergy with hydrolysis promoting DNA bonding, may lead to cancer cell death.

## 1. Introduction

**Scheme 1:**
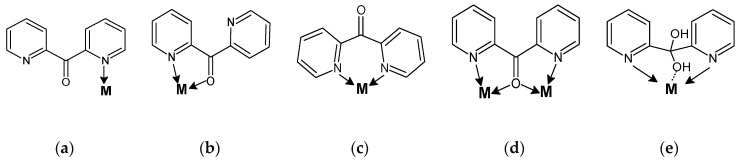
Selected coordination modes of the di-2-pyridylketone (py_2_CO).

Similar to platinum compounds, ruthenium and iridium complexes have properties such as a variety of oxidation states of their central ions, tuneable redox properties, a wide range of coordination numbers and a structural diversity modulated by coordinating ligands, which have been widely studied [1,2]. Such interesting solid-state materials of transition metal complexes have generated potential applications not only in industrial processes as catalysts, photoelectronic sensors, and luminescent chemosensors (in electronic devices), but also in biological and medicinal contexts. Most studies on the biological characterization of platinum, ruthenium and iridium complexes have found them to be useful in medicinal chemistry, especially for chemotherapy. This is one of the forms of treatment of cancer, which is currently a major public health problem. The first anticancer transition-metal complex drug, cisplatin, which was approved by the U.S. Food and Drug Administration in 1978, is the most widely used drug with antineoplastic activity. However, the oncological use of cisplatin is hindered by its severe side effects (nephrotoxicity, neurotoxicity, ototoxicity and nausea) and a very important resistance problem. Resistance develops mainly due to deactivation of the drug by binding to glutathione (GSH) or other sulfur-containing proteins or an increased ability to repair of DNA lesions [2]. Thus, one of the major challenges is looking for new drugs that possess selectivity. To achieve this, ruthenium and iridium complexes have been widely developed as alternatives to anticancer drugs based on platinum(II) complexes (for example: cisplatin [3], carboplatin, oxaliplatin [4], nedaplatin [5], etc.). We have previously reported [6] that, to overcome this problem, our investigation focused on the synthesis of the ruthenium(II) arene complex, [(η^6^-*p*-cymene)RuCl(2,2′-PyBIm)]PF_6_ (2,2′-PyBIm = 2-(2′-pyridyl)benzimidazole). Such ruthenium complexes with a typical half-sandwich structure are well-suited as chemotherapeutic agents because the process of optimizing their activity against cancer cells involves easy modulation of their arene compounds and/or chelating ligands [7]. Unfortunately, while the obtained complex showed moderate activity against the tested carcinoma cells, it was also active against the normal BALB/3T3 cell line (IC_50_ = 43.66 µM; but its cytotoxicity is 15 times lower compared with cisplatin) [6]. Taking this into account, during our following experiments, we tested whether a 2-(2′-pyridyl)benzimidazole replacement with 2,2-bis(4,5-dimethylimidazole) (L^1^) in an arene ruthenium complex {[RuCl(L^1^)(η^6^-*p*-cymene)]PF_6_}·2H_2_O [8] could be a potential chemotherapeutic agent. It displays only moderately cytotoxic activity against the MV-4-11 cell line (IC_50_ = 27.34 µM) in vitro but does not affect the normal BALB/3T3 cell line. This points to the selectivity of the obtained complex. These results motivate our additional investigations. In this paper, we present the synthesis and characterization of not only a new ruthenium(II) arene complex [[(η^6^-*p*-cymene)RuCl(py_2_CO)]PF_6_ (**1**)], but we also extend our studies on the iridium(III) complexes [[(η^5^-Cp)IrCl(py_2_CO)]PF_6_ (**2**) (Cp-pentamethylcyclopentadienyl) and NH_4_[IrCl_4_(py_2_CO)]·H_2_O (**3**)]. Taking into consideration that not only the metal but also the ligands play important roles in their biological activity, we paid special attention to choose a proper chelator possessing N and/or O-donor atoms. We selected for this purpose di-2-pyridylketone (py_2_CO). It should be mentioned that neutral py_2_CO has three potential donor groups, the two 2-pyridyl nitrogen atoms and the carbonyl oxygen atom. In respect to this, this ligand adopts a variety of coordination modes—for example, monodentate (κN), chelating (κ^2^N,N’ or κ^2^N,O), and bridging and chelating (μ_2_-κ^2^N,O:κ^2^O,N’) (Scheme 1a–d) [9]. Moreover, in the presence of water, the ketone carbonyl group can undergo nucleophilic substitution, forming a gem-diol (see Scheme 1e).

The main goal of the present work was: (i) synthesis and full physicochemical characterization of the half-sandwich complexes: 4d^6^ low-spin arene ruthenium(II) (**1**) and (5d^6^) isostructural pentamethylcyclopentadienyl (Cp) iridium(III) (**2**) as well as tetrachloroiridate(III) (**3**) with di-2-pyridylketone (py_2_CO), (ii) determine the structure–activity relationships including not only classical coordination bonds but also noncovalent interactions in a crystal structure, and (iii) investigation of their types of interactions with selected biological targets (DNA, human serum albumin (HSA) and GSH). To achieve these goals, elemental analysis; single-crystal X-ray diffraction; ^1^H, ^13^C, and ^15^N NMR; and FTIR, UV-Vis, PL and circular dichroism (CD) spectroscopy methods were successfully applied. Moreover, the potential cytotoxic effects of **1**–**3** complexes towards the LoVo (colorectal adenoma), MV-4-11 (myelomonocytic leukaemia), MCF-7 (breast adenocarcinoma) and normal BALB/3T3 (mouse fibroblast) cell lines were examined. Taking these findings into consideration, two main mechanisms of action (MoA) are proposed for iridium(III) complexes as a consequence of possible DNA binding according to the hydrolysis of complexes, as well as ROS generation. Additionally, we determined the biocatalytic activity of (**2**, **3**) against NADH (β-nicotinamide adenine dinucleotide disodium salt hydrate).

## 2. Experimental Section

### 2.1. Materials and Physical Measurements

IrCl_3_·xH_2_O (99.8%), [(*p*-cymene)Ru(*μ*-Cl)Cl]_2_ (99%), [CpIr(*μ*-Cl)Cl]_2_ (99%), di-2-pyridylketone (py_2_CO), NH_4_PF_6_ (99%), cisplatin, 5,5′-dithio-bis(2-nitrobenzoic acid) (DTNB) (99%), human serum albumin (HSA) (lyophilized powder, <0.007% fatty acid), L-glutathione reduced form (GSH) (lyophilized powder, >98%), and reduced β-nicotinamide adenine dinucleotide disodium salt hydrate (NADH) were purchased from Sigma-Aldrich Chemie GmbH (Steinheim, Germany).). Analytical grade solvents were purchased from Chempur (Piekary Śląskie, Poland). Tris (hydroxymethyl) aminomethane-HCl (5 mM Tris-HCl/50 mM NaCl, pH = 7.2) and PBS (10 mM, pH 7.4) buffer were prepared using MiliQ water.

Elemental analysis was run on an Elementar Vario Micro Cube analyzer (Elementar, Langenselbold, Germany). The FTIR spectra were recorded on a Nicolet 380 spectrophotometer (Thermo Fisher Scientific, Waltham, Massachusetts, USA) in the spectral range 4000–400 cm^−1^ using the KBr discs method. UV-Vis measurements in ethyl acetate or methanolic solutions were performed on a UV-Vis spectrophotometer (V-630 Jasco, JASCO Corporation, Tokyo, Japan) using 1 cm cuvettes. All absorbance measurements were recorded at room temperature and concentrations 1 × 10^−4^–10^−5^ M for ligand (py_2_CO) and complexes (**1**–**3**). Corrected steady-state luminescence spectra and emission decays were measured by means of Gilden Photonics FluoroSense (Gilden Photonics Ltd., Clydebank, United Kingdom) and FluoroSense-P fluorimeters (Gilden Photonics Ltd., Clydebank, United Kingdom). The latter allows measurements of the emission lifetimes with a temporal resolution of ca. 0.01 μs. Room temperature emission studies were performed in solutions deaerated by prolonged saturation with preliminarily purified and dried argon. The 77 K measurements were performed in fused silica tubes with a 3 mm inner diameter by placing them in a liquid nitrogen filled Dewar flask with a bottom transparent finger. As a quantum yield standard, a solution of quinine sulphate in 0.1 N H_2_SO_4_ (φ_em_ = 0.51) [10] was used. The experimental decay curves were analyzed by the single-curve method using reference convolution based on the Marquardt algorithm [11] with the χ^2^ test and the distribution of residuals serving as the main criterion in the evaluation of the fit quality. Circular dichroism spectra were recorded with a Jasco J-815 spectropolarimeter (Jasco Inc., Kettenbrückengasse, Austria). The NMR spectra were recorded either on Varian VNMRS-500 (Varian Inc., Palo Alto, CA, USA) or Varian VNMRS-600 spectrometers (Varian Inc., Palo Alto, CA, USA) equipped with a 5-mm Z-SPEC Nalorac IDG 500-5HT gradient probe or a 5-mm PFG AutoXID (1H/X15N-31P) probe, respectively. Samples in DMSO-d_6_ solution were recorded at 25 °C, whereas those in D_2_O were recorded at varied temperatures. Standard pulse sequences were used except for the ^1^H-{^15^N} correlation. Gradient-enhanced IMPACT-HMBC [12] ^1^H-{^15^N} correlation spectra were optimized for a coupling constant of 8 Hz with the following experimental conditions: an acquisition time of 0.2 s, spectral windows of 8000 (F2) and 15,000–27,000 (F1) Hz, 1603 complex data points in t2, 128 complex data points in t1, 8 scans per increment, 30 ms WURST-2 mixing sequence centered within the 60 ms preparation interval (ASAP2) and a 140 Ernst angle as the excitation pulse [13]. The data were processed with linear prediction in t1 followed by zero-filling in both dimensions. Gaussian weighting functions were applied in both domains prior to Fourier transformation. All the spectra were referenced according to IUPAC recommendations [14].

### 2.2. Complexes Prepration

#### 2.2.1. [(η^6^-p-cymene)RuCl(py_2_CO)]PF_6_ (1)

A solution (8 mL MeOH and 2 mL CH_2_Cl_2_) of the precursor compound [(η^6^-*p*-cymene)Ru(*μ*-Cl)Cl]_2_ (0.1 mmol, 0.0613 g) was added dropwise to a 8 mL MeOH solution of the ligand di-2-pyridylketone (0.2 mmol, 0.0368 g). Next, to such a mixture, 2 mL of methanol solution of NH_4_PF_6_ (0.2 mmol, 0.0326 g) was added. The dark green reaction mixture was stirred and refluxed for 5 h (~60 °C). After that step, the solution was filtered and allowed to stand at room temperature to crystallize. The resulting brown crystals of complex was filtered off and dried in a vacuum box. Yield 53%. Anal. Calc. (%) for C_21_H_22_ClF_6_PRuN_2_O: C, 42.04; H, 3.70; N, 4.67. Found: C, 42.32; H, 3.43; N, 4.89; IR (cm^−1^): 3091 (w), 2964 (w), 1679 (s), 1592 (m), 1540 (w), 1472 (m), 1436 (m), 1315 (m), 1280 (m), 1238 (w), 1160 (w), 1089 (m), 1058 (w), 944 (w), 839 (s), 755 (m), 67 (m), 557 (s), 478 (w), 461 (w). ^1^H NMR (600 MHz, DMSO-d_6_, δ ppm): 1.14 [6H, d, (C_13/14_**H**_3_)_2_C_12_H)]; 2.53 [1H, m, (C_13/14_H_3_)_2_C_12_**H**)]; 5.90 (2H, C_7**/**7′_**H**, (p-cymene)]; 5.71 (2H, C_8**/**8′_**H**, (p-cymene)]; 1.81 [3H, s, C_10_**H**_3_]; 9.09 [2H, d, C_6/6′_**H**]; 7.96 [2H, d, C_5/5′_**H**]; 8.33 [2H, d, C_4/4′_**H**]; 8.19 [2H, t, C_3/3′_**H**]. ^13^C NMR (150 MHz, DMSO-d_6_, δ ppm): 22.2 (**C**_13_, **C**_14_); 30.6 (**C**_12_); 108.1 (**C**_11_); 85.8 (**C**_7/7′_); 86.2 (**C**_8/8′_); 101.3 (**C**_9_); 17.6 (**C**_10_); 185.3 [**C**_1_, (CO)]; 153.2 (**C**_2/2′_); 127.5 (**C**_3/3′_); 141.0 (**C**_4/4′_); 129.8 (**C**_5/5′_); 157.6 (**C**_6/6′_). ^15^N NMR (60 MHz, DMSO-d_6_, δ ppm): −154.9 (**N**_1/1′_).

#### 2.2.2. [(η^5^-Cp)IrCl(py_2_CO)]PF_6_ (2)

Complex **2** was prepared similarly to complex **1** using [CpIr(*μ*-Cl)Cl]_2_ (0.1 mmol, 0.0797 g) as the metal source. The orange crystals appeared after a week and were collected at 68% yield. Anal. Calc. (%) for C_21_H_23_ClF_6_PIrN_2_O: C, 36.44; H, 3.35; N, 4.05. Found: C, 36.40; H, 3.48; N, 3.91; IR (cm^−1^): 3101 (w), 3077 (m), 2980 (w), 2923 (w), 1681 (s), 1591 (s), 1561 (m), 1491 (m), 1468 (m), 1434 (m), 1382 (w), 1311 (m), 1277 (m), 1245 (m), 1184 (w), 1160 (w), 1098 (w), 1029 (m), 940 (w), 837 (s), 756 (m), 694 (w), 655 (m), 558 (w), 457 (w). ^1^H NMR (600 MHz, DMSO-d_6_, δ ppm): 1.33 [15H, (Cp-C_12-16_**H**_3_)]; 8.93 [2H, d, C_6/6′_**H**]; 7.99 [2H, d, C_5/5′_**H**]; 8.35 [2H, d, C_4/4′_**H**]; 8.25 [2H, t, C_3/3′_**H**]. ^13^C NMR (150 MHz, DMSO-d_6_, δ ppm): 8.1 (Cp-**C**_12-16_H_3_); 89.8 (Cp-**C**_7-11_H_3_); 185.8 [**C**_1_, (CO)]; 152.0 (**C**_2/2′_); 127.7 (**C**_3/3′_); 141.8 (**C**_4/4′_); 131.5 (**C**_5/5′_); 155.4 (**C**_6/6′_). ^15^N NMR (60 MHz, DMSO-d_6_, δ ppm): −167.5 (**N**_1/1′_).

#### 2.2.3. NH_4_[IrCl_4_(py_2_CO)]·H_2_O (3)

A solution of the iridium(III) chloride salt (0.1 mmol, 0.0299 g) in 5 mL of methanol was added dropwise to a 15 mL (CH_3_OH:CH_2_Cl_2_; 3V:1V) mixed solution of the ligand di-2-pyridylketone (0.2 mmol, 0.0326 g) and NH_4_PF_6_ (0.25 mmol, 0.0407 g). The reaction mixture was stirred and refluxed for 10 h (~60 °C). After that step, the solution was filtered and allowed to stand at room temperature to crystallize. The resulting complex was filtered off and dried in a vacuum box after a week. The violet crystals were collected at 75% yield. Anal. Calc. (%) for C_11_H_13_Cl_4_IrN_3_O_2_: C, 23.88; H, 2.37; N, 7.59. Found: C, 23.79; H, 2.69; N, 7.35; IR (cm^−1^): 3502 (bm), 3198 (s), 3096 (s), 1622 (m), 1589 (w), 1566 (w), 1513 (s), 1458 (w), 1429 (s), 1410 (s), 1347 (s), 1273 (w), 1257 (s), 1287 9w), 1150 (w), 1108 (w)1085 (w), 1055 (w), 1034 (w), 997 (m), 967 (w), 822 (m), 759 (m), 731 (w), 687 (m), 669 (s), 623 (w), 455 (w). ^1^H NMR (600 MHz, DMSO-d_6_, δ ppm): 9.55 [1H, d, C_3_**H**]; 8.15 [1H, d, C_4_**H**]; 8.01 [1H, d, C_5_**H**]; 9.61 [1H, d, C_6_**H**]; 8.29 [1H, d, C_3′_**H**]; 8.21 [1H, d, C_4′_**H**]; 7.77 [1H, d, C_5′_**H**]; 8.90 [1H, d, C_6′_**H**]; 7.04 (N**H**_4_^+^). ^13^C NMR (150 MHz, DMSO-d_6_, δ ppm): 210.0 [**C**_1_, (CO)]; 155.6 (**C**_2_); 136.6 (**C**_3_); 138.5 (**C**_4_); 132.4 (**C**_5_); 153.1 (**C**_6_); 150.0 (**C**_2′_); 128.0 (**C**_3′_); 138.8 (**C**_4′_); 130.4 (**C**_5′_); 149.7 (**C**_6′_). ^15^N NMR (60 MHz, DMSO-d_6_, δ ppm): −160.3 (**N**_1_); −66.6 (**N**_1′_), −356.2 (**N**H_4_^+^).

### 2.3. Crystal Structure Determination

Single crystal X-ray diffraction data of compounds **1** and **3** were collected at 120(2) K on a Stoe IPDS-2T diffractometer with graphite-monochromated Mo-Kα radiation. Data collection and image processing were performed with X-Area 1.75 (STOE and Cie GmbH, Darmstadt, Germany, 2015) [15]. Intensity data were scaled with LANA (part of X-Area) in order to minimize differences in intensities of symmetry-equivalent reflections (multi-scan method). The crystal was thermostated in nitrogen stream at 120 K using CryoStream-800 device (Oxford CryoSystem, Long Hanborough OX29, UK) during the entire experiment. Owing to the high absorption coefficients (8.916 mm^−1^), numerical absorption corrections were applied based on measured crystal faces. The structures of **1**, **3** were solved with the ShelXT [16] structure solution programs run under Olex2 [17] using Intrinsic Phasing and refined with the ShelXL [18] refinement package. The WinGX [19] program was used to prepare the final version of CIF files. Diamond [20] was used to prepare the figures. Non-hydrogen atoms were refined with anisotropic displacement parameters. Positions of the C–H and O–H hydrogen atoms were calculated geometrically and taken into account with isotropic temperature factors and refined as constrained, using the AFIX 43 (for –CH groups) and AFIX 6 (for O–H) instructions. The H atoms in ammonium ions were located in a difference map and refined with the N–H distances restrained to 0.91(2) Å. In the compound **3**, residual electron density is somewhat high and localizes near the heavier Ir(III) ion. In our opinion, it is an artefact (e.g., due to cut of Fourier series), since no atom can be present at that location. A summary of crystallographic data is shown in Table 1. A representative diffraction image from single-crystal X-ray diffraction data of complex **3** is given in the Supplementary Materials Figure S1. Crystallographic data for the structure of **1** and **3** reported in this paper have been deposited with the Cambridge Crystallographic Data Centre as supplementary publications No. CCDC 2004801 and 2004802. Copies of the data can be obtained free of charge on application to CCDC, 12 Union Road, Cambridge CB2 1EZ, UK (Fax: (+44) 1223-336-033; Email: deposit@ccdc.cam.ac.uk).”

### 2.4. Cell Viability Assays

The cytotoxicity of the investigated complexes **1**–**3** was determined by microculture with sulforhodamine B SRB (all adherent cells) and MTT ([3-(4,5-dimethyl(thiazol-2-yl) -2,5-diphenyltetrazoliumbromide)]) (for cells in suspension (leukemia cells)) assays with the following three tumor cell lines: colorectal adenoma (LoVo), myelomonocytic leukemia (MV-4-11), and breast adenocarcinoma (MCF-7), as well as a healthy mouse fibroblast (BALB/3T3) cell line. The cytotoxicity of the compounds was measured using the methods described previously [21]. The in vitro cytotoxic activity results were presented as the percentage of inhibition of cell proliferation by the tested compounds or DMSO, which was used as a solvent (at a concentration of 10 µg/mL of the tested compound was 0.1% DMSO). The results for cisplatin, used as a test control, were presented in the form of IC_50_ value (this is the concentration of the compound causing the inhibition of cell proliferation of 50% of the cell population). The results were calculated from 2 to 3 independent experiments.

### 2.5. Lipophilicity, CT-DNA, HSA and GSH Interactions with Analysed Complexes

Determination of log P values, and studies on the interactions of ruthenium(II) (**1**), iridium(III) complexes (**2**, **3**) with CT-DNA, HSA and GSH, were performed according to the previous procedure published in the paper [21].

### 2.6. DNA Melting Tests

The melting profiles of DNA/complex mixtures were recorded on a Jasco J-815 spectropolarimeter (Kettenbrückengasse, Austria) equipped with a CDF-426S/15 Peltier temperature controller. CD melting experiments were performed using 100 μM CT DNA concentration, in the absence and presence of 100 equivalents of complexes (in Tris-HCl/NaCl buffer, pH 7.2). CD melting were performed at 5 °C/min heating rate in the 35–95 °C temperature range. Each spectrum was recorded in the 220–300 nm wavelength range, averaged over three scans and subtracting the buffer baseline. After each temperature change, the system was left to equilibrate for 3 min. From 3D data, we selected the proper wavelength (256 nm) and obtained a classic 2D melting profile (fraction folded vs. temperature). The melting temperatures (T_m_) were mathematically calculated by using the curve fitting function in Origin 2020 software (OriginLab, Northampton, MA, USA). T_m_ values represent the melting temperature of the DNA with and without complexes.

### 2.7. Reaction with NADH

The reaction of analyzed complexes (1 µM) with NADH (100 µM) in 50% MeOH/50% H_2_O (V:V) was examined by UV-Vis spectroscopy at 37 °C after various time intervals (0.5–9 h). The turnover number (TON) was calculated from the difference in NADH concentration after 9 h divided by the concentration of analyzed iridium complexes. The concentration of NADH was obtained using the extinction coefficient ε_339_ = 6220 M^−1^cm^−1^.

## 3. Results and Discussion

**Scheme 2:**
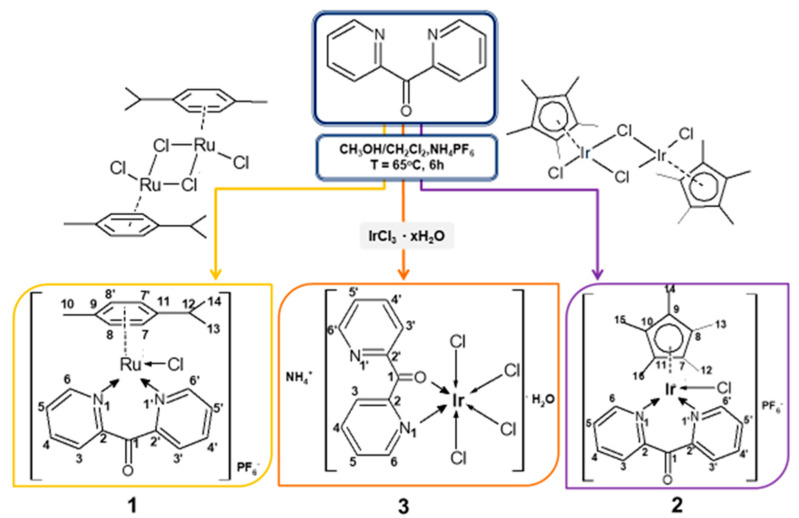
Synthesis procedures for complexes **1**-**3** including the numbering of the relevant atoms for the assignment of the appropriate to NMR data.

The iridium complexes [(η^5^-Cp)IrCl(py_2_CO)]PF_6_ (**2**) and NH_4_[IrCl_4_(py_2_CO)]·H_2_O (**3**) were obtained using two methods of synthesis: the reaction of metal precursor [(η^5^-Cp)Ir(*μ*-Cl)Cl]_2_ or metal salt (IrCl_3_·xH_2_O) with 2-dipyridilketone (py_2_CO), respectively. Details connected with synthetic conditions of complexes preparation are shown in Scheme 2. The similar half-sandwich Ru(II) complex **1** was obtained in the same procedure as complex **2** but a ruthenium source was adopted [(η^6^-p-cymene)Ru(*μ*-Cl)Cl]_2_. All the complexes were found to be stable in air and non-hydroscopic. These complexes are soluble in polar solvents, such as methanol, DMSO and nonpolar ethyl acetate.

### 3.1. Description of the Structures

**Figure 1:**
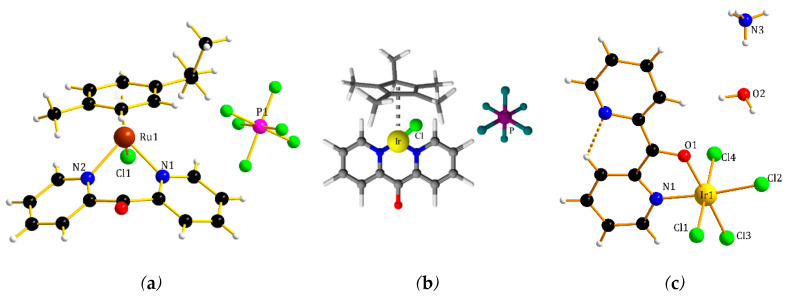
Molecular structures of complexes: (**a**) **1**; (**c**) **3**; (**b**) **2** with proposed structure of complex.

As mentioned in the introduction, di-2-pyridylketone adopts a variety of coordination modes, which is demonstrated in the structure of the obtained complexes. In complex **1**, the py_2_CO-donor binds to the Ru(II) ion in a bidentate manner (κ^2^N,N’). The pseudo-octahedral geometry of the half-sandwich complex is completed by the *p*-cymene moiety and a chlorido ligand (Figure 1a).

**Figure 2:**
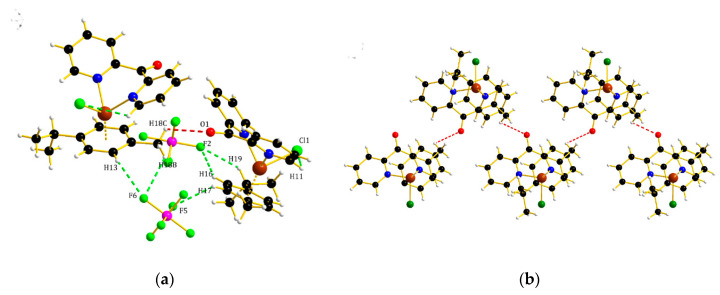
The short contacts for complex **1** (**a**) and (**b**) fragment of the molecular structure of **1** showing the zig-zag motif view the c direction.

The Ru···Cg (Cg denotes the gravity ring center of C(12)–C(13)–C(14)–C(15)–C(16)–C(17)) distance (1.6841(12) Å) (Table 2) as well as the Ru–Cl distance (2.3942(4) Å) and the Ru-N (2.1022(14) and 2.0950(14) Å) bond lengths are similar to other ruthenium complexes [8]. The six-membered chelate ring Ru(1)–N(1)^∩^N(2) adopted a boat conformation with the Ru(1) and C(6) atoms below the plane at 0.986 and 0.420 Å, respectively. The crystal packing analysis revealed the collection of the intra- and intermolecular short contacts shown in Figure 2 and listed in Table 3.

**Figure 3:**
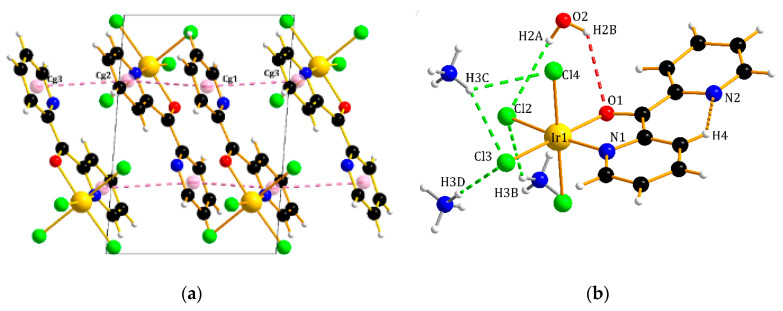
(**a**) The π···π interactions (aqua molecule and ammonium ion are omitted for clarity), (**b**) intra- and intermolecular hydrogen-bond interactions of **3**.

Unfortunately, using the same protocol for the preparation of crystals for **2**, we were unable to obtain proper monocrystals for X-ray measurement. However, comparative analysis of the ^15^N NMR data and FTIR spectra confirmed the same coordination mode of the py_2_CO ligand in the suggested [(η^5^-Cp)IrCl(py_2_CO)]PF_6_ (**2**) complex. On the other hand, we noticed in the molecular structure of **3** a rare chelating κ^2^N,O coordination mode. Such coordination is caused by the formation of a strong intramolecular C(4)–H(4)···N(2) hydrogen bond owing to the rotation of the C6–C7 bond connected with the uncoordinated pyridine fragment (Figure 1c). The double bond character of the C=O group of the py_2_CO ligand in **3** is confirmed by its proper bond length (1.2828(13) Å), which is typical for the C=O bond, being in a range of 1.21–1.23 Å [22]). In addition, the coordination sphere of Ir(III) with a distorted octahedral geometry is completed by four Cl^−^ ligands. The structural parameters in the neutral fragment are normal compared with those in the IRIM and ICR-type complexes [23,24]. The lengths of the Ir–Cl bonds are in the range 2.329(2)–2.354(2) Å, while the lengths of the bonds with nitrogen and oxygen donors are significantly shorter and reach Ir(1)–N(1) 1.998(7) Å and Ir(1)–O(1) 2.053(6) Å, respectively. The deviation from the ideal angles in the coordination sphere of Ir(III) is 9.4° in the case of the formation of a six-membered chelate ring by organic ligand N(1)-Ir(1)-O(1) 79.2(2) (Table 2). The molecules of **3** are stabilized in the crystal structure through proper arrangement of hydrogen bonds, such as N–H···O, N–H···Cl, O–H···O, and C–H···N, along with π···π interactions (Figure 3 and Table 3). Two symmetry-related layer structures are stacked in a slipped face-to-face mode and contacted through π···π (Cg1···Cg2 distance of 3.8043(9) Å and Cg1···Cg3 distance of 3.7387(10) Å) interactions to form a double layer (Figure 3a).

### 3.2. Spectral Studies

#### 3.2.1. ^1^H, ^13^C and ^15^N NMR

Bearing in mind that the structure of complex **2** is not confirmed by SC X-ray data, the ^1^H, ^13^C and ^15^N NMR spectral studies are required; therefore, the formation of **2** and **1**, **3** was deduced from the obtained NMR data too. The selected spectra are depicted in Figures S2, S3a, S4a and S5a in the Supplementary Material; the ensuing data are summarized in the experimental section, and some of the data are presented in Table 4.

Multinuclear magnetic resonance spectroscopy (^1^H, ^13^C, ^15^N) was applied to determine the environment of the central atom and the mode of ligand binding method in complexes **1–3**. The comparison of the appropriate chemical shifts for the complex (δ_complex_) and the free ligands (δ_ligand_) enables calculating all the coordination shifts (Δ_coord._ = δ_complex_ – δ_ligand_), which can help to suggest the structure in a solution. Considering the ^13^C and ^15^N NMR results, the coordination mode of py_2_CO in a solution was confirmed in **1**–**3** complexes in accordance with SC X-ray data. It was found that all the resonance signals corresponding to nitrogen atoms in py_2_CO were shifted after coordination to metal ion (Table 4). In both the [(η^6^-*p*-cymene)RuCl(py_2_CO)]PF_6_ (**1**) and [(η^5^-Cp)IrCl(py_2_CO)]PF_6_ (**2**) compounds, the py_2_CO-donor binds Ru(II) and Ir(III) ion in a bidentate manner (κ^2^N,N’), while in complex **3**, Ir(III) is coordinated in rare chelating κ^2^N,O manner. Additionally, ^1^H NMR confirmed that coordination by N’-donating atom is blocked by formation of strong C–H···N hydrogen bond (large Δ_coord._ = +0.95 ppm).

#### 3.2.2. FTIR Spectra

The infrared spectrum of the di-2-pyridylketone showed characteristic bands due to ν(C=O) 1683 cm^−1^, ν(C=N) 1429 cm^−1^ and ν(C=C) 1581 cm^−1^ (see Figure S6 and Table S1). The ν(C=O) band in the spectra of **1** and **2** complexes was only slightly shifted towards lower frequencies (1679 and 1681 cm^−1^, respectively), indicating that the ligand is not coordinated to the metal centers through a keto-carbonyl group. As we expected, di-2-pyridylketone acted as a chelating N,N-donor, confirmed by stretching vibrations ν(C=N) shifted to 1436 (**1**) or 1434 cm^−1^ (**2**). On the other hand, while comparing the spectra of the free py_2_CO with the NH_4_[IrCl_4_(py_2_CO)]·H_2_O (**3**) complex, we observed that ν(C=O) stretching vibrations were significantly shifted to lower wavenumbers (1622 cm^−1^, Δ = 61 cm^−1^), which pointed to a coordination of the oxygen atom of the keto group to the iridium(III) center. Additionally, this complex showed two intense bands at approximately 1429 and 1410 cm^−1^, which are assigned to stretching bands of the nitrogen atoms from pyridine rings. In consequence, only the nitrogen of one pyridine molecule of py_2_CO takes part in coordination to the metal center formed together with the oxygen atom (C=O) chelating ring. The spectral data obtained for complexes **1** and **3** are in agreement with the SC-X-ray structures and for complex **2** confirmed by ^15^N NMR data.

#### 3.2.3. Electronic Spectra

**Figure 4:**
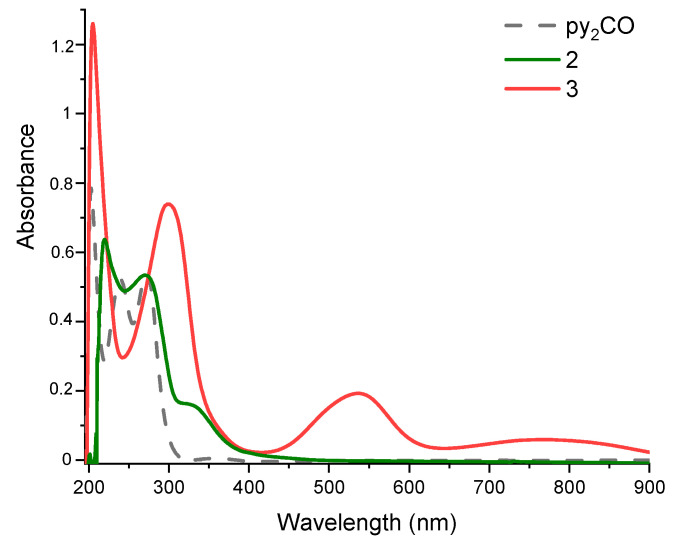
UV-Vis spectra for **2** and **3** (solid) along with py_2_CO (dashed) in ethyl acetate.

To provide correct UV-Vis analysis of the obtained complexes in solution, we presented electronic spectra in the same solution for the ligand (py_2_CO) (Figure 4 and Figure S7). Additionally, to compare the influence of nonpolar and polar solvents on the UV-Vis spectra of the complexes, we measured their spectra in ethyl acetate and methanol, respectively.

Comparing the free ligand spectrum with the electronic spectra of **1**–**3** complexes in methanol shows the main bands corresponding to the ligand centered in the π→π* and n→π* transition (see Table 5). Additionally, upon coordination of py_2_CO with metal ions, the weaker π→π* absorption bands are present as the slope of the strongest band at approximately 250 nm. Additionally, in the spectra of complex **3** in both solvents is observed a band at 536 (509) nm (shoulder ca. 773, 744 nm), associated with molar absorptivity of 3569 (1167) M^−1^ cm^−1^. This band, according to the κ^2^N,O coordination mode of py_2_CO in complex **3**, can be described as MLCT transition involving nonbonding and antibonding combinations of the metal d and p orbitals of oxygen atom (C=O). This kind of absorption is not observed in the UV-Vis spectra of complexes (**1**, **2**), displaying the κ^2^N,N coordination mode of py_2_CO. It should be underlined that the band corresponding to the ^3^T_1g_←^1^A_1g_ transition for metal complexes d^6^ (ground state ^1^*I*)) in octahedral geometry (^1^A_1g_ (t_2g_^6^)) is both Laporte and spin forbidden. Moreover, in the polar solvent, we observed a redshift of UV bands arising from π→π * transitions and a blue shift of a n→π* transition in the range 299–360 nm.

**Figure 5:**
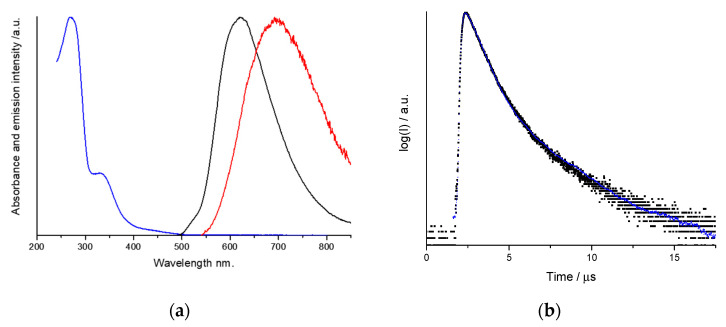
(**a**) Band profile of UV-Vis absorption spectrum for [(η^5^-Cp)IrCl(py_2_CO)]PF_6_ (**2**) complex recorded in dichloromethane solution (blue line). Band profiles of emission spectra recorded at room temperature in dichloromethane solution (red line) and in 77 K MeOH/EtOH 1:1 matrices (black line). (**b**) Emission decay profiles recorded for complex **2** (black scattered line) and the fitted decay curve (black scattered line). Data for the 77 K measurements in MeOH/EtOH 1:1 matrices.

On the other hand, iridium complexes are notable for their interesting luminescence properties. Therefore, Ir(III) complexes with a proper ligand have found application as efficient photosensitizers for PDT, as a probe of ions, molecules and organelles [25,26,27]. In our experimental conditions, the [(η^5^-Cp)IrCl(py_2_CO)]PF_6_ (**2**) complex exhibited intense yellow-orange photoluminescence in frozen (77 K) MeOH/EtOH 1:1 mixture (Figure 5).

For complex **2** (λ_em_ = 620 nm), bi-exponential decay was observed with two life-time values, with a short-lived (τ_1_ = 0.02 (0.07) μs) and long-lived component (τ_2_ = 0.83 (0.93) μs), respectively. Bi-exponential decays with two τ_em_ values were fitted according to the I_em_(t) = A_1_ exp(−t/τ_1_) + A_2_ exp(−t/τ_2_) relationship. Values given in parentheses next to the life-time values correspond to the contribution of the short-lived A_1_τ_1_/(A_1_τ_1_ + A_2_τ_2_) and the long-lived A_2_τ_2_/(A_1_τ_1_ + A_2_τ_2_) components of the overall emission quantum yields. The short-lived emission component occurs with very small contribution to the overall emission intensity, whereas the long-lived component is predominant. The data for a solution of **2** in acetonitrile or dichloromethane at room temperature displayed the emission band ca. 700 nm with low quantum yields (ϕ_em_) equal to 0.03% or 0.04%, respectively. Complex **3** at 77 K in alcohol glass exhibited very weak and structured emission for which it was impossible to estimate the τ_em_ value. Notably, compared to complex **2** with the κN,N’ coordination mode of py_2_CO, the emission of **3** is very weak, probably due to the directly bonded oxygen (of C=O) with the iridium ion in the κN,O mode of coordination of py_2_CO.

### 3.3. Biological Activity of the Complexes

#### 3.3.1. Solution Stability and Lipophilicity

**Figure 6:**
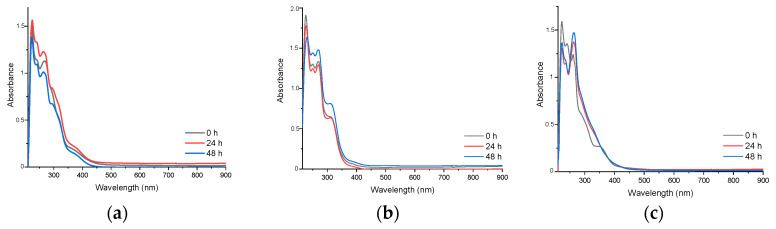
Time-dependent stability studies on complexes: (**a**) **1**; (**b**) **2** and (**c**) **3** in Tris-HCl/NaCl buffer solution monitored by UV–Vis absorption spectra.

The stability of complexes in solution and lipophilicity play a crucial role in the process of developing a new drug. In our experiment, the UV-Vis and ^1^H NMR spectroscopies were used over a period of 48 h to monitor the stability of the complexes in a Tris-HCl/NaCl buffer and DMSO, respectively. In the spectra of complex **2** (Figure 6), we observed decreasing intensity of spectral bands, while in spectra of complexes **1**, **3** the characteristic bands are slightly red shifted (e.g., 223 →225 nm, 261→263 nm, respectively).

**Figure 7:**
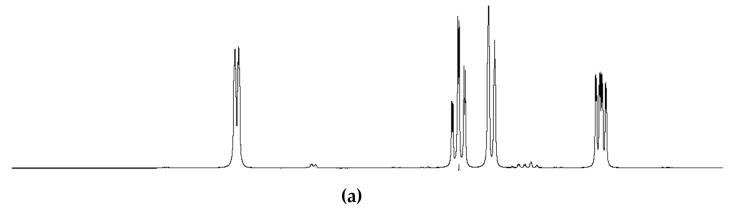

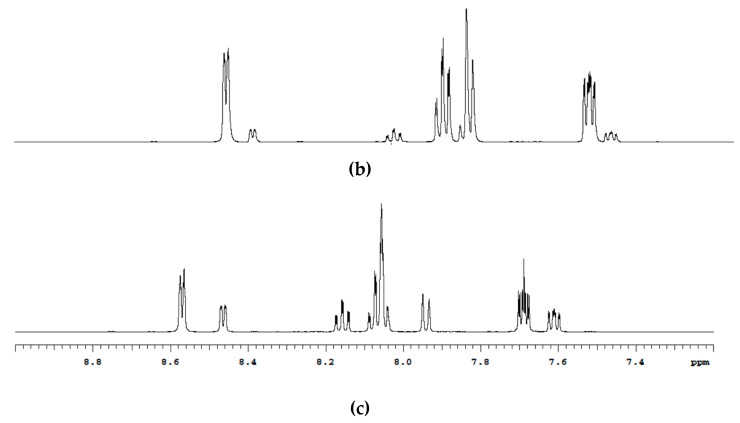
500 MHz ^1^H NMR spectra of a solution (**a**) containing 15 mg of py_2_CO dissolved in 0.5 mL of D_2_O (99.8%), (**b**) solution (a) + 10 μL of 5% DCl/D_2_O, (**c**) solution (**b**) + 25 μL of 5% DCl/D_2_O, showing changes in the equilibrium of the keto carbonyl group (py_2_CO) to gem-diol (py_2_CO·H_2_O).

Such changes suggest that the process of hydrolysis takes places under these conditions. We additionally confirmed this process using the ^1^H NMR method (see Figures S3–S5). Hydrolysis of the obtained complexes besides chloride ions is also caused by the presence in the structure of di-2-pyridylketone. This ligand has been observed to undergo hydration of the keto carbonyl group forming a gem-diol (py_2_CO·H_2_O) [9]. In practice, there is often an equilibrium between hydrated form and ketones in an aqueous solution, although the equilibrium usually lies far over towards the ketone form [9]. This was detected by the ^1^H and ^13^C NMR method used for hydration reaction of py_2_CO with D_2_O (Table 6 and Figure 7).

**Figure 8:**
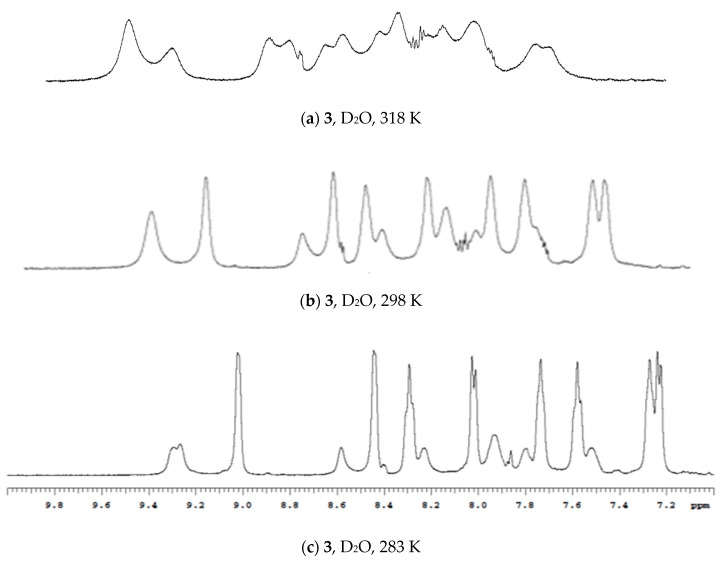
500 MHz ^1^H NMR spectrum of a solution containing 5 mg of **3** dissolved in 0.5 mL of D_2_O (99.8%), showing temperature dependence of the complex hydrolysis.

During the coordination reaction, this process of py_2_CO hydrolysis is favored by metal ions, so in the literature complexes with the gem diol coordinating ligands [9] are presented. For the hydrolysis of the obtained complexes, both the substitution of chloride ions as well as hydration of keto carbonyl group (py_2_CO) should be taken into consideration. The effective process of hydrolysis of complex **3** is illustrated by ^1^H and 2D NMR spectra in D_2_O (Table 6, Figure 8, Figures S8–S10).

Bearing in mind that the lipophilicity of potential anticancer compounds has a major impact on their accumulation in cells and especially their penetration through cell membranes, we determined the log *P* values of the analyzed complexes. As examined, the **1**–**3** complexes are hydrophilic; however, complexes **1** and **2** in the cationic form (log *P* = −1.44 ± 0.11 (**1**), −1.14 ± 0.06 (**2**)) are more hydrophilic than complex **3** (log *P* = −0.30 ± 0.05). According to a predictive model, compounds with intermediate lipophilicity (log *P* between −2 and 2 with an optimum at 0) tend to accumulate in the mitochondria of tumor cells [28]. However, the problem of the target of the complexes still needs further detailed research.

#### 3.3.2. Cell Viability Studies and Interactions with GSH

**Figure 9:**
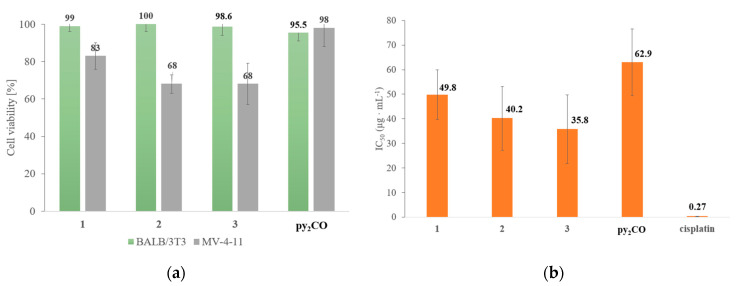
Effect of complexes **1**–**3** and free py_2_CO on viability of normal cell line BALB/3T3 and cancer cell line MV-4-11 (**a**), IC_50_ values of the analyzed compounds and cisplatin against MV-4-11 (**b**).

The complexes and free py_2_CO were tested for in vitro cytotoxicity against the MV-4-11, MCF-7, LoVo cancerous cell lines and non-tumorigenic mouse fibroblasts (BALB/3T3). Only a weak cytotoxic effect against the LoVo (**1**) and MCF-7 (**2**) cell lines was observed (inhibition of cell proliferation at a concentration of 10 μg/mL equal to 1.4 % and 3.7 %, respectively). The cytotoxicity profiles of the complexes towards healthy fibroblasts (BALB/3T3) (Figure 9a) pointed towards the selectivity of the obtained **1**–**3** complexes. On the other hand, the in vitro cytotoxicity studies (cell viability with compound concentration 10 μg/mL) against MV-4-11 revealed the moderate activity of **1**–**3** complexes (Figure 9a). Additionally, for this line, Figure 9b illustrates the data of the half maximal inhibitory concentration (IC_50_) for the researched complexes. Taking into consideration the selectivity of the obtained complexes and a detailed analysis of their cytotoxicity, we should carefully select proper cancer cell lines for future experiments.

To see if the presence of GSH significantly reduces the cytotoxic activity of the complexes, glutathione deactivation experiments were carried out using the Ellman method [21,29]. The results given as the concentration of unreacted GSH remaining after 28 h incubation with the iridium(III) complexes show that both iridium(III) complexes **2** and **3** had the same results (0.037 mmol/L). Comparing similar data of cell viability and IC_50_ for both iridium(III) complexes with GSH deactivation results, some correlation was observed. It is worth noting that the result for cisplatin is equal to 0.034 mmol/L, suggesting that this complex binds with high affinity to GSH.

#### 3.3.3. DNA and HSA-Binding Studies

It is commonly believed that the main target for antitumor metal complexes is DNA, as has been shown for platinum/ruthenium drugs [30]. In terms of the antitumor activity of iridium complexes, this is probably the consequence of possible DNA binding according to the hydrolysis of the complexes, as well as ROS generation or interactions with proteins. Therefore, in our work, we paid particular attention to research into the nature of the antitumor activity of the analyzed complexes.

##### DNA-Complex Interactions Monitored by UV-Vis and CD

**Figure 10:**
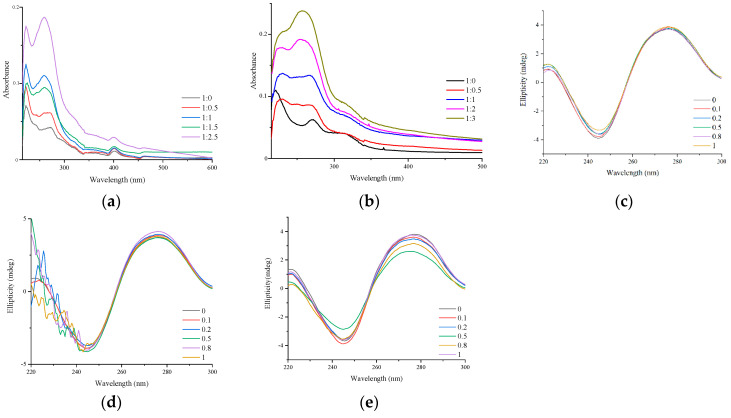
(**a**,**b**) UV-Vis spectra of **1**, **2** (5 × 10^−6^ M) in Tris buffer solution with increasing concentration of CT-DNA (0–1.5 × 10^−5^ M) at room temperature; (**c**–**e**): CT-DNA-circular dichroism (CD) spectra (CT-DNA 1.5 × 10^−4^ M) after 24 h incubation at different [DNA]/[complex] ratios at 37 °C.

Absorption titration studies were conducted to investigate a possible binding mode and intrinsic equilibrium binding constant (K_b_) for the complexes with CT-DNA by monitoring the change in absorbance at various concentrations of DNA. As shown in Figure 10, the spectra of cationic complexes **1** and **2** show increases in absorbance without shifts of maxima at a wavelength of approximately 401 and 315 nm, respectively. Those changes are indicative of non-covalent and non-intercalative modes of complex binding to CT-DNA [31,32] with K_b DNA_ = 1.43 × 10^5^ and 1.28 × 10^5^ (linear plot for K_b_ determination see Figure S11). However, the spectral data obtained for **3** suggest ambiguous binding interaction between the complex and CT-DNA. Therefore, the above inconclusive results were additionally verified by circular dichroism measurements (CD). The CD spectra of **3** showed hypochromism with a decrease in intensity for both the negative ellipticity band at 245 nm and the positive ellipticity band at 280 nm. After an overrun molar ratio of 0.5, we observed hyperchromism with a isodichroic point (257 nm), as shown in Figure 10e. Thus, this suggests that two possibilities for complex **3**–CT-DNA interactions should be considered [31,33,34,35], because the metal complex can act as a dual-function complex. They can bind to DNA both through intercalation of the planar py_2_CO ligand and by the metal coordination to the DNA bases.

##### DNA Melting Studies Assessed by 3D CD

**Figure 11:**
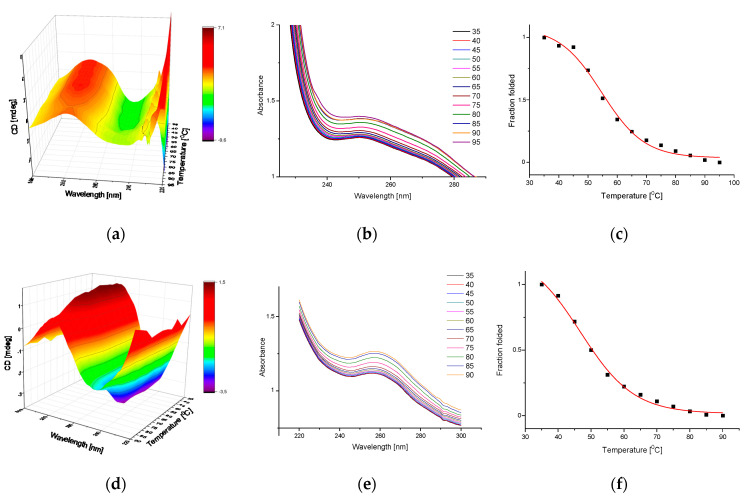
Three-dimensional CD melting of CT-DNA (100 μM) in Tris-HCl/NaCl at pH 7.2 with complexes **2** (**a**–**c**) and **3** (**d**–**f**), 2D absorbance spectra at 256 nm and calculated fraction folded as a function of temperature.

To solve the problem of the mode of iridium-DNA binding, covalent and non-covalent, DNA melting experiments [36] were conducted. Considering the higher sensitivity of circular dichroism, 3D CD spectroscopy was used. The T_m_ of the tested solution was determined as the transition midpoint of the melting curve by monitoring the maximum of the ellipticity and absorbance as a function of temperature ranging from 35 to 95 °C. Figure 11 shows changes in the CD spectrum with increasing temperature of the mixture of CT-DNA and complexes **2**, **3**. We noted an increase in the CD band at 282 nm and a decrease in the negative band at 242 nm. The thermal denaturation profiles showed differences between iridium complexes that we attributed to the stronger influences of complex **3** on the stability of CT-DNA. The melting temperature (T_m_) of free CT-DNA (55.2 ± 0.7 °C; Figure S12) decreased by approximately 0.5 and 8.5 °C after the addition of **2**, **3**, respectively. According to the literature [37,38], a slight shift or no shift in the T_m_ value of a DNA-complex mixture suggests non-classical binding modes, such as electrostatic-binding along the phosphate backbone of DNA, groove-binding and/or surface-binding modes. This outcome confirmed the results for complex **2** interactions with CT-DNA that were observed via the UV-Vis and CD spectroscopy. The relatively large negative ΔT_m_ value reflects that complex **3** induced destabilization of the DNA double helix, as has been previously noticed for cisplatin and other complexes [39,40]. This effect is caused by the probable formation of covalent cross-links.

#### 3.3.4. Reaction of Iridium Complexes with NADH as Source of ROS

**Figure 12:**
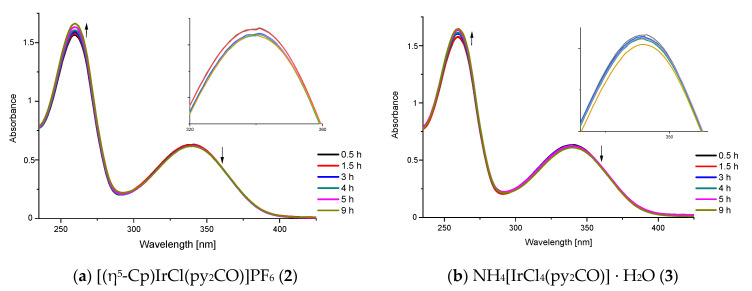
UV-Vis spectra for the catalytic oxidation of NADH (100 μM) to NAD^+^ in the presence of complexes (1 μM) over a 9 h period in 50% MeOH/50% H_2_O (V:V) (inset: wavelength absorbance changes from 315-360 nm).

According to the literature, iridium complexes can oxidize NADH by hydride transfer and produce reactive oxygen species, thus providing a pathway for the oxidation mechanism of action and increase its anticancer activity. In our work, the catalytic ability of the analyzed iridium complexes was monitored as it decreased from its maximum at 339 nm, which corresponds to the conversion of NADH to NAD^+^ (Figure 12). Based on these data, the turnover numbers (TONs) of the complexes were calculated as follows: 4.8 NH_4_[IrCl_4_(py_2_CO)]·H_2_O (**3**) > 3.3 [(η^5^-Cp)IrCl(py_2_CO)]PF_6_ (**2**). Compared to iridium complex **2**, complex **3** showed a stronger ability to interfere with coenzyme NAD^+^/NADH coupling through the transfer of hydrogenation reactions and to induce ROS in cells, which is also consistent with their anticancer activities. As mentioned above, in contrast to the opinion that DNA is the main target for platinum/ruthenium compounds, other authors claimed that DNA independent mechanisms such as inhibition of metalloproteinases, interference with adhesion processes, and increased levels of reactive oxygen species (ROS) are responsible for their antitumor and antimetastatic activity of iridium complexes.

#### 3.3.5. HSA-Complex Interactions via UV-Vis and CD Spectroscopy

**Figure 13:**
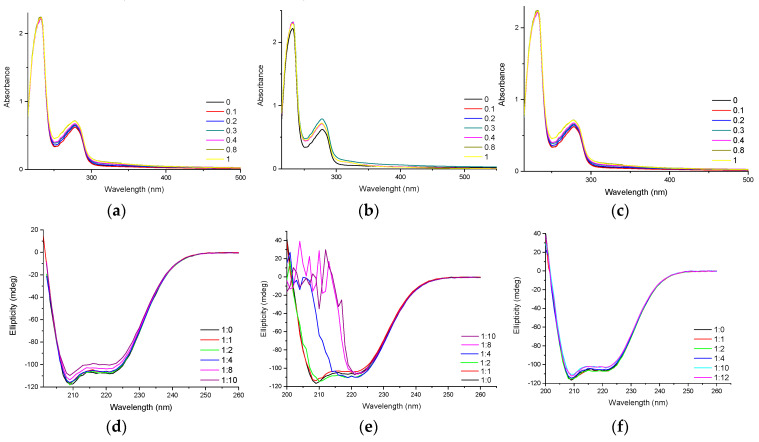
Changes in UV-Vis absorption spectra of HSA (2 × 10^−5^ M) in Tris-HCl/NaCl buffer upon addition of **1** (**a**), **2** (**b**) and **3** (**c**) in different molar ratio and CD spectra of HSA incubated with **1** (**d**), **2** (**e**) and **3** (**f**) in different molar ratio.

Because plasma albumins can interfere with anticancer compounds and change their biological activity, especially their cytotoxicity effectiveness, it is important to obtain insight into interactions of the analyzed complexes with HSA as a plasma protein. The UV-Vis spectra of HSA with increasing amounts of complexes show increasing of HSA absorbance without any shift (Figure 13 a–c). The binding constants (3.22 × 10^5^ (**1**), 1.52 × 10^5^ (**2**) and 6.54 × 10^4^ (**3**)) and spectra changes may suggest interactions between HSA and the complexes. To detect any changes of the microenvironment or the conformations of HSA caused by the analyzed complexes, CD spectroscopy was used (Figure 13e,f). According to the abovementioned K_b_, the α-helical structure of HSA under the influence of Ru(II) (**1**) or Ir(III) (**2**, **3**) complexes changed from 56% to 51%, 52% and 55%, respectively. The noticeable, spectral changes indicate a partial unfolding of the protein structure, leading to the loss of its stability.

## 4. Conclusions

In summary, three di (2-pyridyl) ketone-containing complexes with a Ru(II) or Ir(III) ion were synthesized and characterized by different methods, and the solid-state crystal structures of complex **1** and **3** were established. Spectro-analytical techniques showed that the complexes adopted an octahedral geometry through coordinated *p*-cymene (**1**) or Cp (**2**), one molecule of py_2_CO and chloride ions. The chelating ligand adopts two coordination modes: κN,N’ (**1**, **2**) or rare κN,O (**3**). The stability of the complexes in solution demonstrates that the presence of di-2-pyridylketone and chloride ions in the structure promotes a hydrolysis process. This ligand has been observed to undergo hydration of the keto carbonyl group, forming a gem-diol (py_2_CO·H_2_O). In vitro cytotoxic studies against the MV-4-11, MCF-7, LoVo cell lines and non-tumorigenic mouse fibroblasts (BALB/3T3) were performed. The most promising results were obtained for iridium(III) complex **3** against MV-4-11 (IC_50_ = 35.8 ± 13.9 µg/mL) without any toxic effect on normal BALB/3T3, which points to its selectivity as a potential anticancer agent. The preliminary insight into their mode of binding with DNA achieved via the UV-Vis and CD spectroscopy confirmed for **1**, **2** cationic complexes non-classical binding modes, such as electrostatic-binding, groove-binding and/or surface-binding modes. Meanwhile, the 3D CD experiment (ΔT_m_) reflects that complex **3** induced the probable formation of covalent bonds with DNA. Furthermore, complex **3** showed a stronger ability to interfere with coenzyme NAD^+^/NADH, which is coupled to the induction of ROS in cells, which is also consistent with their anticancer activities (Figure 7c). According to the obtained data, the better antiproliferative activity of the iridium(III) complexes **2** and **3** was derived from a synergistic effect of MoA (hydrolysis and redox reactions generating ROS).

## Figures and Tables

**Table 1:** Crystal data and structure refinement for **1** and **3**.

**Empirical Formula**	C_21_H_22_ClF_6_PRuN_2_O	C_11_H_14_Cl_4_IrN_3_O_2_
**Formula Weight**	599.89	554.27
**Temperature**	120(2) K	120(2) K
**Wavelength**	0.71073 Å	0.71073 Å
**Crystal System, Space Group**	Monoclinic, P 2_1_/c	Triclinic, *P-1*
**Unit Cell Dimensions**	a = 8.5232(2) Å, α = 90 deg. b = 24.3326(5) Å, β = 91.523(2) deg. c = 10.8425(2) Å, γ = 90 deg.	a = 7.2392(18) Å, α = 84.42(2) deg. b = 9.933(3) Å, β = 75.32(2) deg. c = 11.721(4) Å, γ = 84.38(2) deg.
**Volume**	2247.85(8) A^3^	809.1(4) A^3^
**Z, Calculated Density**	4, 1.773 Mg/m^3^	2, 2.275 Mg/m^3^
**Absorption Coefficient**	0.954 mm^−1^	8.916 mm^−1^
**F(000)**	1200	524
**Theta Range for Data Collection**	2.390 to 25.500 deg.	3.028 to 25.497 deg.
**Limiting Indices**	−10 ≤ h ≤ 10, −29 ≤ k ≤ 29, −13 ≤ l ≤ 13	−8 ≤ h ≤ 8, −12 ≤ k ≤ 12, −14 ≤ l ≤ 14
**Reflections Collected/Unique**	23359/4173 [R(int) = 0.0278]	6480/2997 [R(int) = 0.0639]
**Completeness to Theta**	25.242–99.8%	25.242–99.4%
**Refinement Method**	Full-matrix least-squares on F^2^	
**Data/Restraints/Parameters**	4173/0 301	2997/19/209
**Goodness-of-fit on F^2^**	1.102	1.037
**Final R Indices [I > 2sigma(I)]**	R_1_ = 0.0209, wR_2_ = 0.0546	R_1_ = 0.0501, wR_2_ = 0.1326
**R Indices (all Data)**	R_1_ = 0.0213, wR_2_ = 0.0548	R_1_ = 0.0531, wR_2_ = 0.1364
**Largest diff. Peak and Hole**	0.550 and −0.412 e·A^−3^	2.568 and −2.835 e·A^−3^

**Table 2:** Bond lengths [Å] and angles [deg.] for **1** and **3**.

[(η^6^-p-cymene)RuCl(py_2_CO)]PF_6_ (**1**)					
Ru(1)–N(1)	2.1022(14)	N(1)–Ru(1)–N(2)	84.54(6)	–	–
Ru(1)–N(2)	2.0950(14)	N(1)–Ru(1)–Cl(1)	86.44(4)	–	–
Ru(1)–Cl(1)	2.3942(4)	N(2)–Ru(1)–Cl(1)	85.83(4)	–	–
Ru(1)···Cg	1.6841(12)	–	–	–	–
NH_4_[IrCl_4_(py_2_CO)]·H_2_O (**3**)					
Ir(1)–N(1)	1.998(7)	N(1)–Ir(1)–O(1)	79.2(2)	Cl(3)–Ir(1)–Cl(2)	89.94(8)
Ir(1)–O(1)	2.053(6)	N(1)–Ir(1)–Cl(3)	97.0(2)	Cl(1)–Ir(1)–Cl(2)	90.49(11)
Ir(1)–Cl(1)	2.354(2)	O(1)–Ir(1)–Cl(3)	175.61(15)	N(1)–Ir(1)–Cl(4)	90.4(2)
Ir(1)–Cl(2)	2.349(2)	N(1)–Ir(1)–Cl(1)	89.1(2)	O(1)–Ir(1)–Cl(4)	87.62(17)
Ir(1)–Cl(3)	2.329(2)	O(1)–Ir(1)–Cl(1)	92.35(17)	Cl(3)–Ir(1)–Cl(4)	90.26(8)
Ir(1)–Cl(4)	2.354(2)	Cl(3)–Ir(1)–Cl(1)	89.74(8)	Cl(1)–Ir(1)–Cl(4)	179.56(6)
–	–	N(1)–Ir(1)–Cl(2)	173.07(19)	Cl(2)–Ir(1)–Cl(4)	89.63(8)
–	–	O(1)–Ir(1)–Cl(2)	93.88(17)	–	–

**Table 3:** Hydrogen bonds for **1** and **3** [Å and deg.] and π···π interactions parameters.

D–H···A	d(D–H)	d(H···A)	d(D···A)	<(D–H···A)
[(η^6^-p-cymene)RuCl(py_2_CO)]PF_6_ (**1**)				
C(18)–H(18)#1···O(1)	0.96	2.87	3.2172(23)	102.3
C(11)–H(11)···Cl(1)	0.93	2.90	3.2895(18)	106.8
C(19)–H(19)···F(2)	0.98	2.45	3.2735(22)	141.1
C(16)–H(16)···F(2)	0.93	2.49	3.295(2)	144.7
C(17)–H(17)···F(5)#1	0.96	2.52	3.1309(21)	123.2
C(13)–H(13)#1···F(6)#1	0.93	2.57	3.0691(21)	114.1
C(18)–H(18B)#1···F(6)	0.96	2.85	3.2066(22)	102.8
NH_4_[IrCl_4_(py_2_CO)]·H_2_O (**3**)				
C(4)–H(4)···N(2)	0.95	2.27	2.902(11)	123.5
O(2)–H(2B)···O(1)	0.87	2.75	3.403(18)	131.4
O(2)–H(2A)···Cl(2)	0.87	2.82	3.593(14)	148.7
N(3)–H(3B)···Cl(2)#1	0.89(2)	2.89(7)	3.475(9)	125(7)
N(3)–H(3D)···Cl(3)#2	0.89(2)	2.433(3)	3.291(8)	164(8)
N(3)–H(3C)···Cl(3)#3	0.89(2)	2.61(5)	3.396(8)	148(8)
N(3)–H(3C)···Cl(4)#3	0.89(2)	2.71(7)	3.389(8)	134(7)
N(3)–H(3A)···O(2)	0.89(2)	2.01(3)	2.897(17)	174(9)
π···π	d(Cg···Cg)		offset	
Cg1-Cg2#3	3.8043(9)		1.560	
Cg1-Cg3#2	3.7387(10)		1.907	

Symmetry transformations used to generate equivalent atoms (**1**) #1 x, -y+3/2, z-1/2; (**3**) #1 -x+1, -y+1, -z+2; #2 x, y+1, z; #3 -x+2, -y+1, -z+2; Cg1 denotes the gravity ring centre of N(2)-C(7)-C(8)-C(9)-C(10)-C(11), Cg2 and Cg3 denotes the gravity ring center of N(1)-C(1)-C(2)-C(3)-C(4)-C(5).

**Table 4:** ^1^H, ^13^C and ^15^N NMR chemical shifts of **1**–**3** complexes in DMSO-d_6_. The coordination shifts are shown in parentheses *.

Compound	δH6	δC6	δC2	CO	δN1	δN1′	δCp
**1**	9.09 (+0.43)	157.6 (+8.2)	153.2 (−1.5)	185.3 (−8.8)	**−154.9** **(−92.1)**	**−154.9**	–
**2**	8.93 (+0.27)	155.4 (+6.0)	152.0 (−2.7)	185.8 (−8.3)	**−167.5** **(−114.7)**	**−167.5**	89.8 (−2.7)
**3**	9.61 (+0.95)	153.1 (+3.7)	155.6 (+0.9)	**210.0** **(+15.9)**	**−160.3** **(−97.5)**	−66.6 (−3.8)	–
**py_2_CO**	8.66	149.4	154.7	194.1	−62.8	–	–

* Δ_coord._ = δ_complex_ – δ_ligand_; Cp_(Ir)_-η^5^-pentamethylcyclopentadienyl.

**Table 5:** Summary of electronic spectral data of **1**–**3** complexes and free ligand.

Compound	UV-Vis λ/nm (ε (dm^3^/mol·cm))						
	Solvent	ν(π→π*)		ν(n→π*)		MLCT	
**py_2_CO**	**EtOAc**	203 (15299)	239 (10166)	272 (10491)	360 (106)	–	–
	**MeOH**	209 (4488)	240 (8794)	269 (9643)	350 (51)	–	–
**1**	**MeOH**	204 (10857)	249 (4186)	279 (5170)	358 (1027)	–	–
**2**	**EtOAc**	220 (14297)	–	269 (11993)	331 (3531)	–	–
	**MeOH**	224 (17154)	250 (9336)	271 (9026)	323 (3511)	–	–
**3**	**EtOAc**	205 (23272)	–	–	299 (13658)	536 (3569)	773 (1103)
	**MeOH**	220 (10552)	240 (6449)	269 (7412)	291 (6864)	509 (1167)	744 (176)

**Table 6:** ^1^H, ^13^C NMR chemical shifts of ligand (py_2_CO) and complex **3** in D_2_O.

Atom	py_2_CO		3
	D_2_O, δ [ppm]	D_2_O/DCl, δ [ppm]	278 K, D_2_O, δ [ppm]
	Product of Hydrolysis		
**C2**	152.2	156.1	168.5
**H3/C3**	7.79/125.8	7.95/123.3	7.18/124.6
**H4/C4**	7.87/138.3	8.17/143.6	7.52/138.3
**H5/C5**	7.51/127.9	7.62/126.1	7.21/126.1
**H6/C6**	8.44/148.7	8.47/144.5	8.96/150.7
**CO**	194.2	93.3	107.6
**C2′**	–	–	156.4
**H3′/C3′**	–	–	7.97/125.3
**H4′/C4′**	–	–	8.23/147.1
**H5′/C5′**	–	–	7.68/126.6
**H6′/C6′**	–	–	8.38/139.6

## Supplementary Materials

The following are available online at https://www.mdpi.com/1996-1944/13/16/3491/s1, Figure S1. A representative diffraction image from single-crystal X-ray diffraction data (a) of complex 3 (b) zoom; Figure S2: The HMBC ^15^N NMR spectra of complex 1 (a), 2 (b), 3 (c); Figures S3–S5: Time course of changes in the ^1^H NMR spectra of appropriate complex in DMSO-d_6_; measurements were taken (a) immediately after sample preparation and (b) after 24 h. Figure S6: FTIR spectra of free ligand py_2_CO and complex 1 (a), 2 (b) and 3 (c); Table S1: Some important FTIR absorption frequencies for py_2_CO and **1**–**3** complexes *; Figure S7. UV-Vis spectra of free ligand and analysed complexes in methanol and ethyl acetate; Figure S8. ^1^H-^13^C HMBC spectrum of 3 in D_2_O at 278 K. Figure S9. ^1^H-^13^C HSQC spectrum of 3 in D_2_O at 278 K; Figure S10. ^1^H-^1^H NOESY NMR spectrum of complex 3 in D_2_O at low temperature (278 K) showing large exchange cross-peaks for being in equilibrium the main hydrolysis product; Figure S11. Binding constant determination. Plots of [DNA]/(ε_a_-ε_f_) versus [DNA] (a) 1 and (b) 2; Figure S12. (a) 3D CD melting of free CT-DNA (100 μM) in Tris-HCl/NaCl at pH 7.2, (b) 2D absorbance spectra at 256 nm and (c) fraction folded as function of temperature.
