# Supplementary material for: Ruthenium(II) and Iridium(III) Complexes as Tested Materials for New Anticancer Agents
Source: Materials (Basel). 2020 Aug 7;13(16):3491. doi: 10.3390/ma13163491 (PMC7475896; doi:10.3390/ma13163491)
Supplement: Supplementary file 1 [file materials-13-03491-s001.pdf]

materials

Supplementary Material

Ruthenium(II) and iridium(III) complexes as tested materials for new anticancer agents

Joanna Masternak, Agnieszka Gilewska, Iwona Łakomska, Barbara Barszcz, Katarzyna Kazimierczuk, Jerzy Sitkowski, Joanna Wietrzyk, Anna Kamecka and Magdalena Milczarek

---

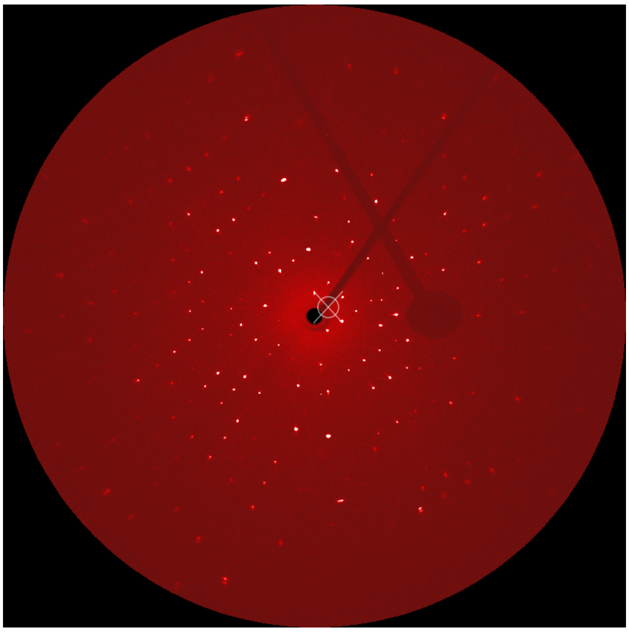

a)

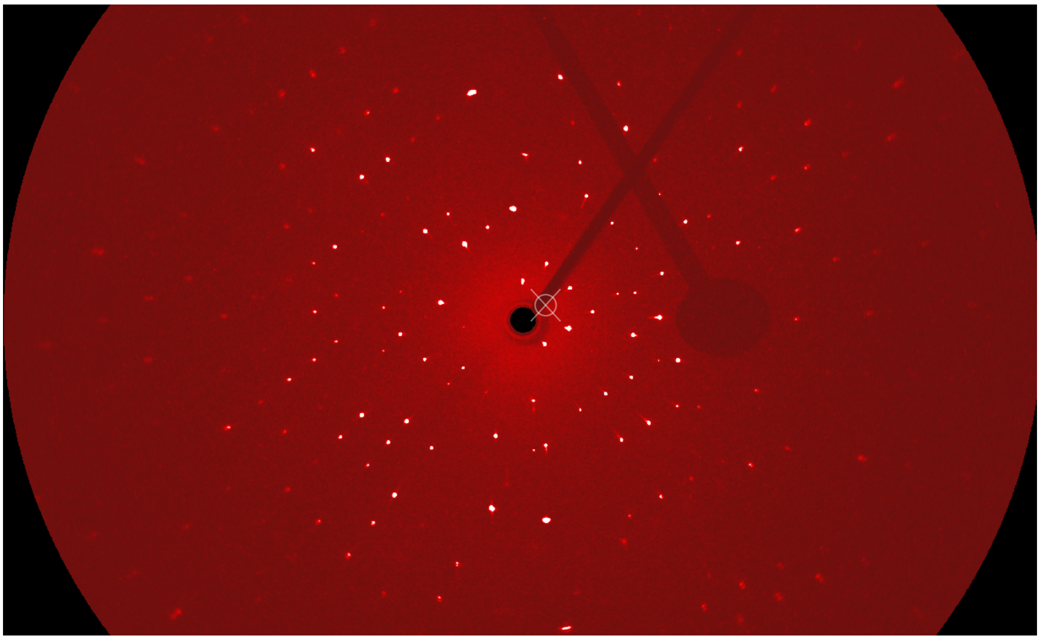

b)

Figure 1. A representative diffraction image from single-crystal X-ray diffraction data a) of complex **3** b) zoom.

a)

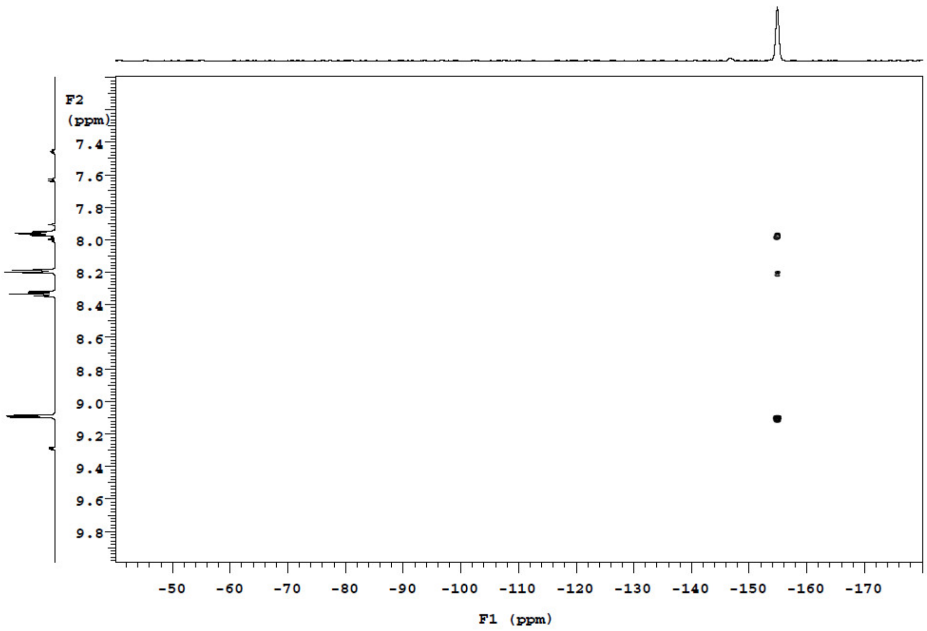
F2
(ppm)
7.4
7.6
7.8
8.0
8.2
8.4
8.6
8.8
9.0
9.2
9.4
9.6
9.8
-50
-60
-70
-80
-90
-100
-110
-120
-130
-140
-150
-160
-170
F1 (ppm)

b)

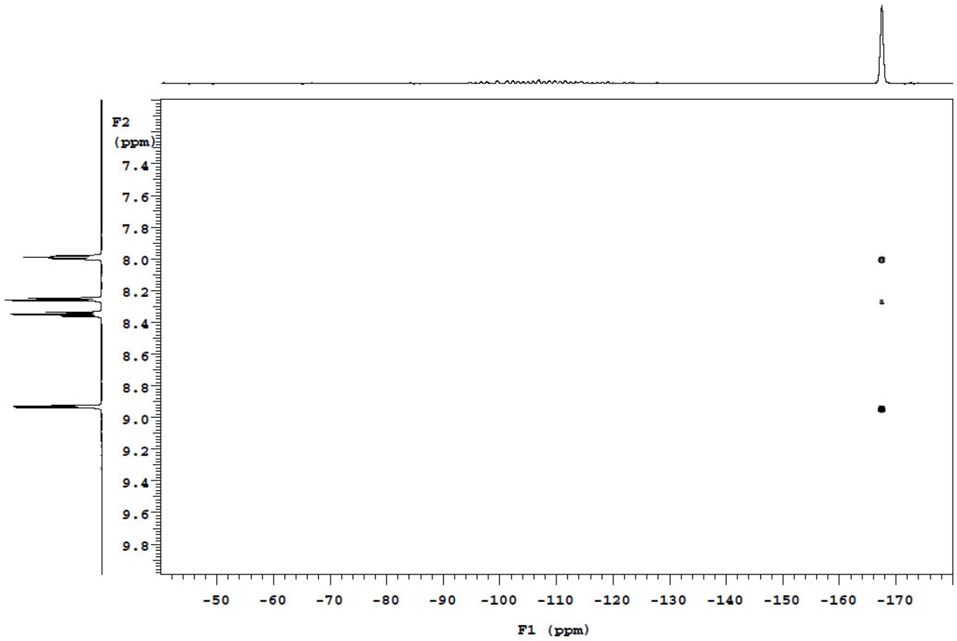
F2
(ppm)
7.4
7.6
7.8
8.0
8.2
8.4
8.6
8.8
9.0
9.2
9.4
9.6
9.8
-50
-60
-70
-80
-90
-100
-110
-120
-130
-140
-150
-160
-170
F1 (ppm)

c)

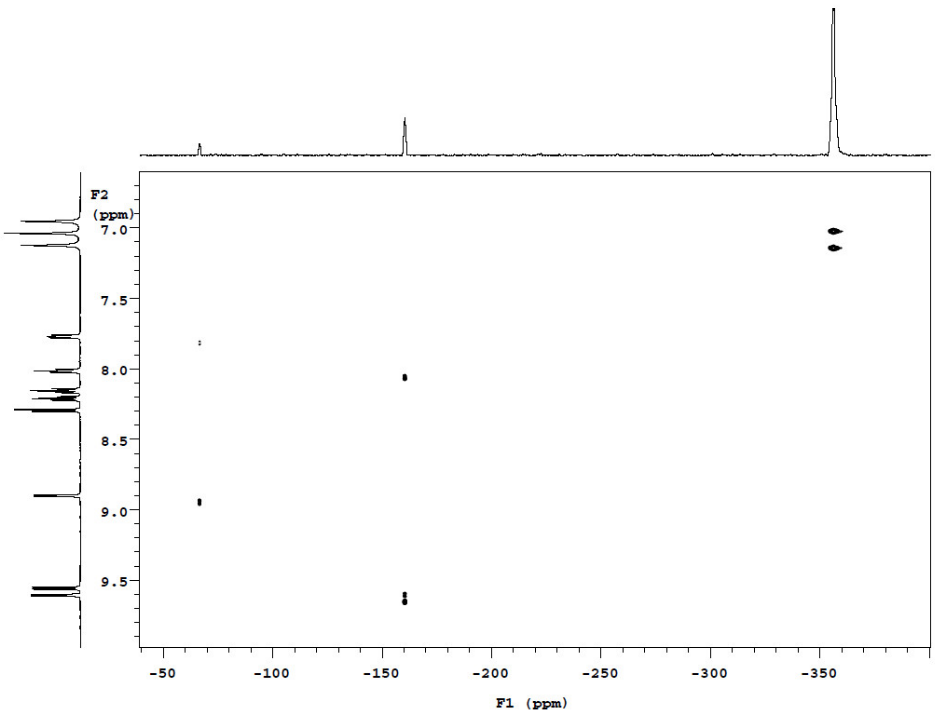

Figure 2. The HMBC ^{15}N NMR spectra of complex **1** (a), **2** (b), **3** (c).

a)

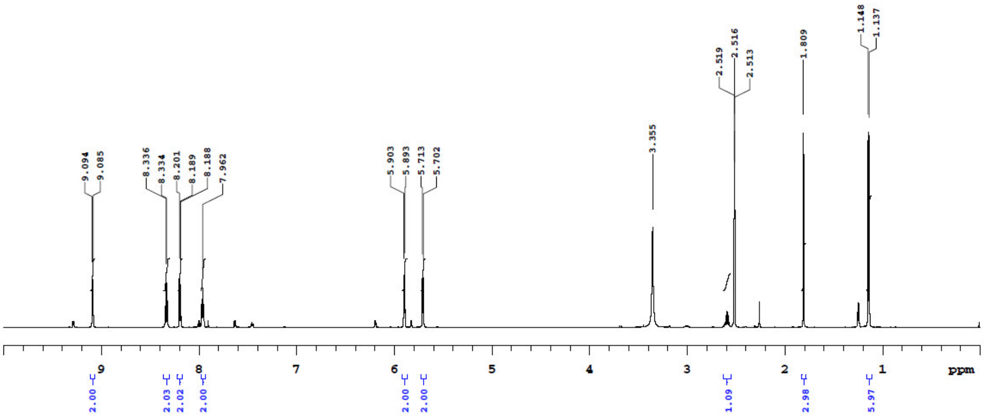

b)

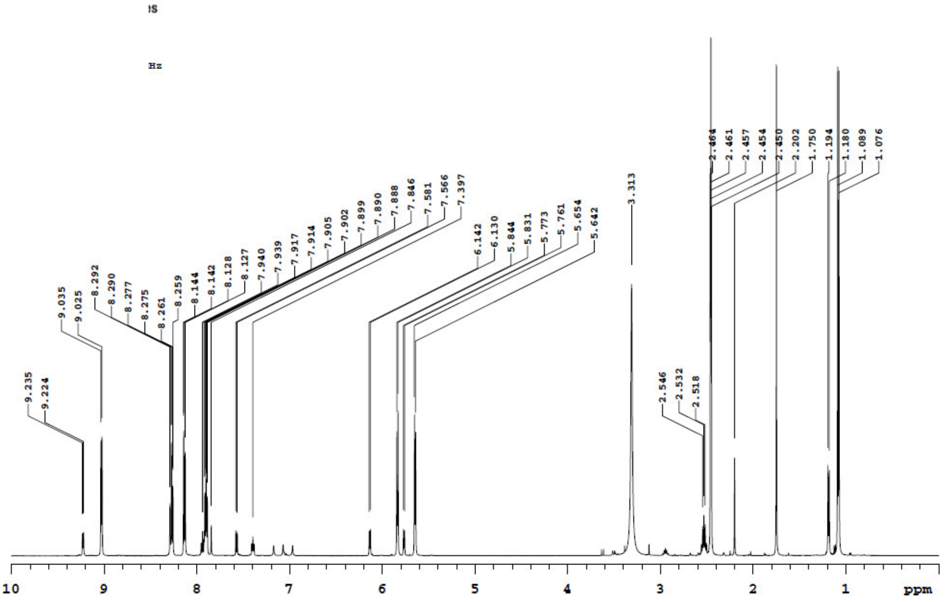

Figure 3. Time course of changes in the ^{1}H NMR spectra of complex **1** in DMSO-d_6; measurements were taken (a) immediately after sample preparation and (b) after 24 h.

a)

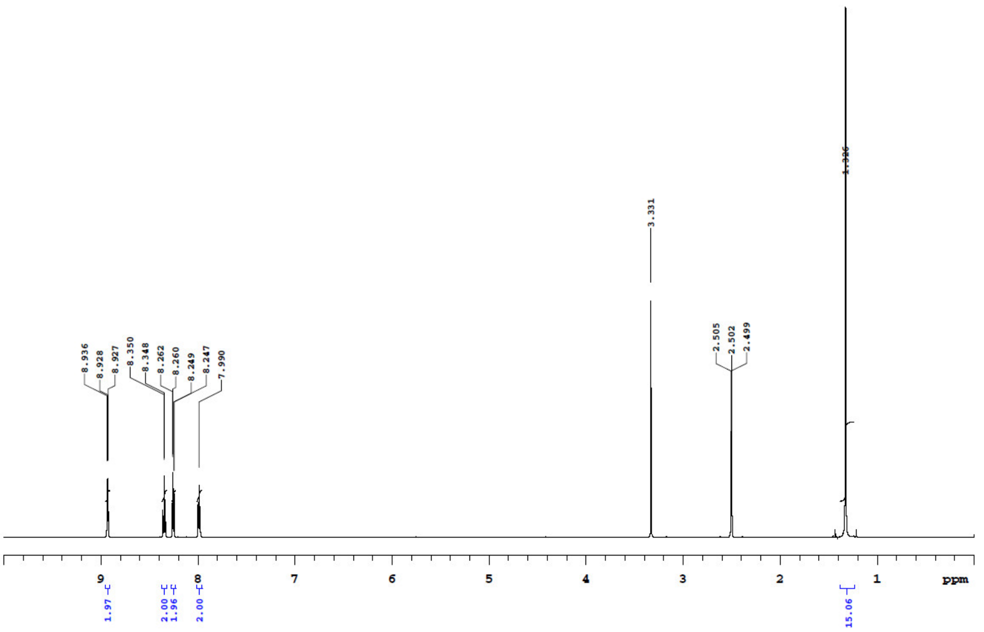

b)

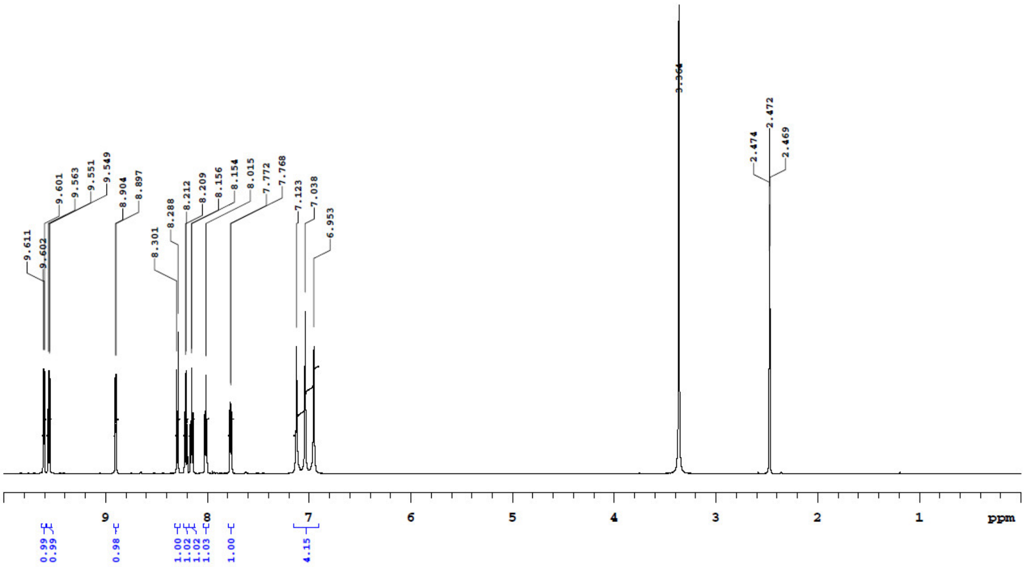

Figure S4. Time course of changes in the ^{1}H NMR spectra of complex **2** in DMSO-d_6; measurements were taken (a) immediately after sample preparation and (b) after 24 h.

a)

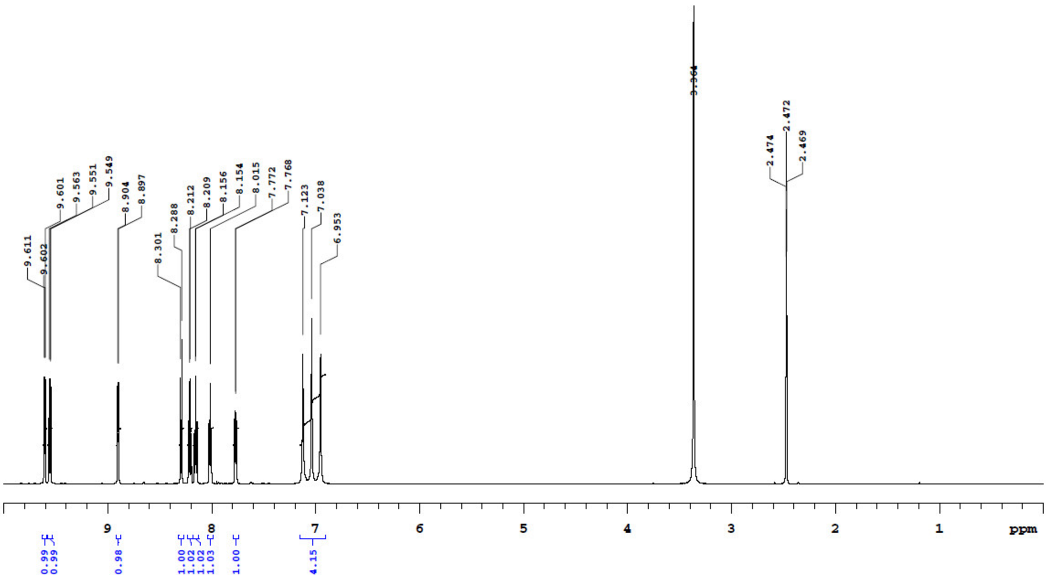

b)

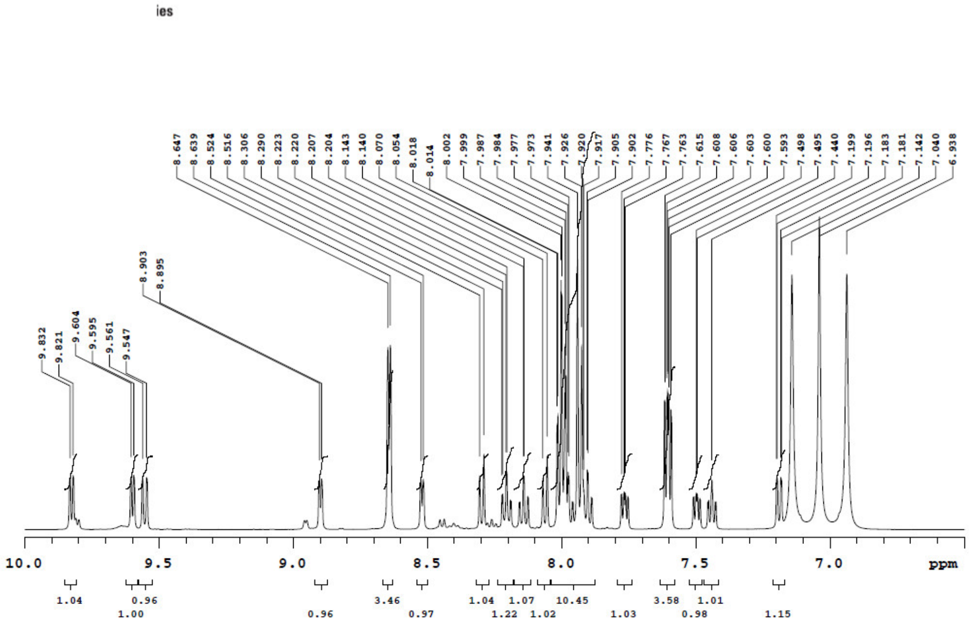

Figure 5. Time course of changes in the ^{1}H NMR spectra of complex **3** in DMSO-d_6; measurements were taken (a) immediately after sample preparation and (b) after 24 h.

a)

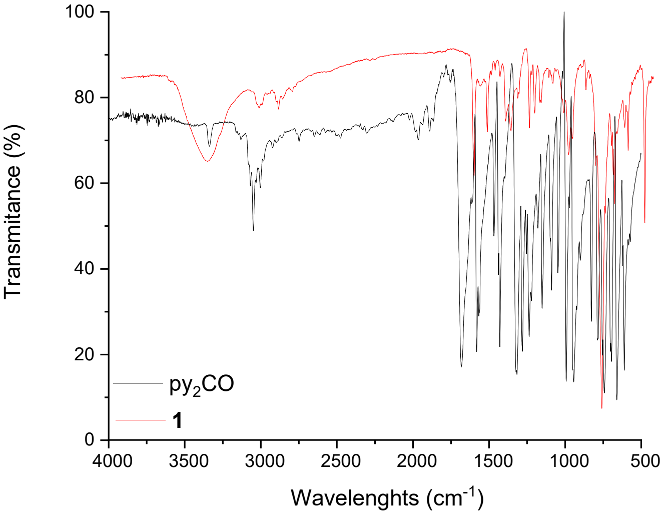

b)

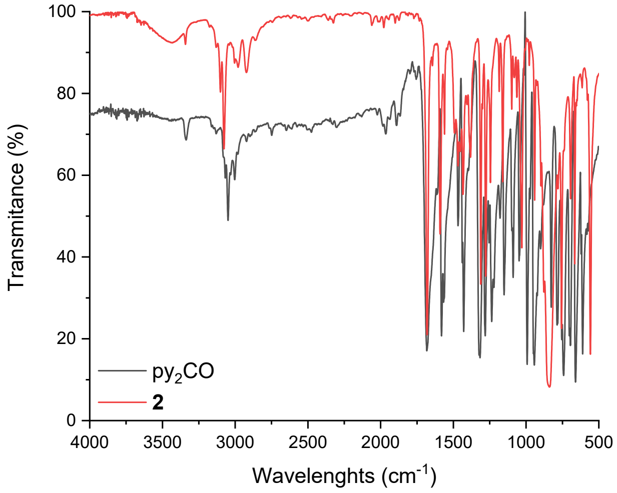

c)

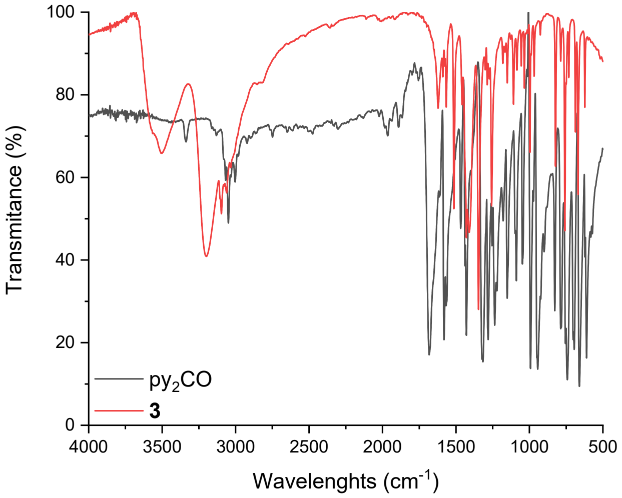

Figure 6. FTIR spectra of free ligand py$_2$CO and complex **1** (a), **2** (b) and **3** (c).

Table 1. Some important FTIR absorption frequencies for py$_2$CO and **1-3** complexes*.

Compound	ν(H_2O)	ν(C=O)	ν(C=C)	ν(C=N)	ν(PF_6^-)	ν(NH_4^+)
py$_2$CO	-	1683(s)	1581(s)	1429(s)	-	-
[(η^6-*p*-cymene)RuCl(py$_2$CO)]PF$_6$ (**1**)	-	1679(s)	1592(m)	1436(m)	839 (s)	-
[(η^5-Cp)IrCl(py$_2$CO)]PF$_6$ (**2**)	-	1681(s)	1591(m)	1434(m)	837(s)	-
NH$_4$[IrCl$_4$(py$_2$CO)] · H$_2$O (**3**)	3502	1622(m)	1566(m)	1429(s) 1410(m)	-	3198(s)

* s - strong, m - medium, w - weak;

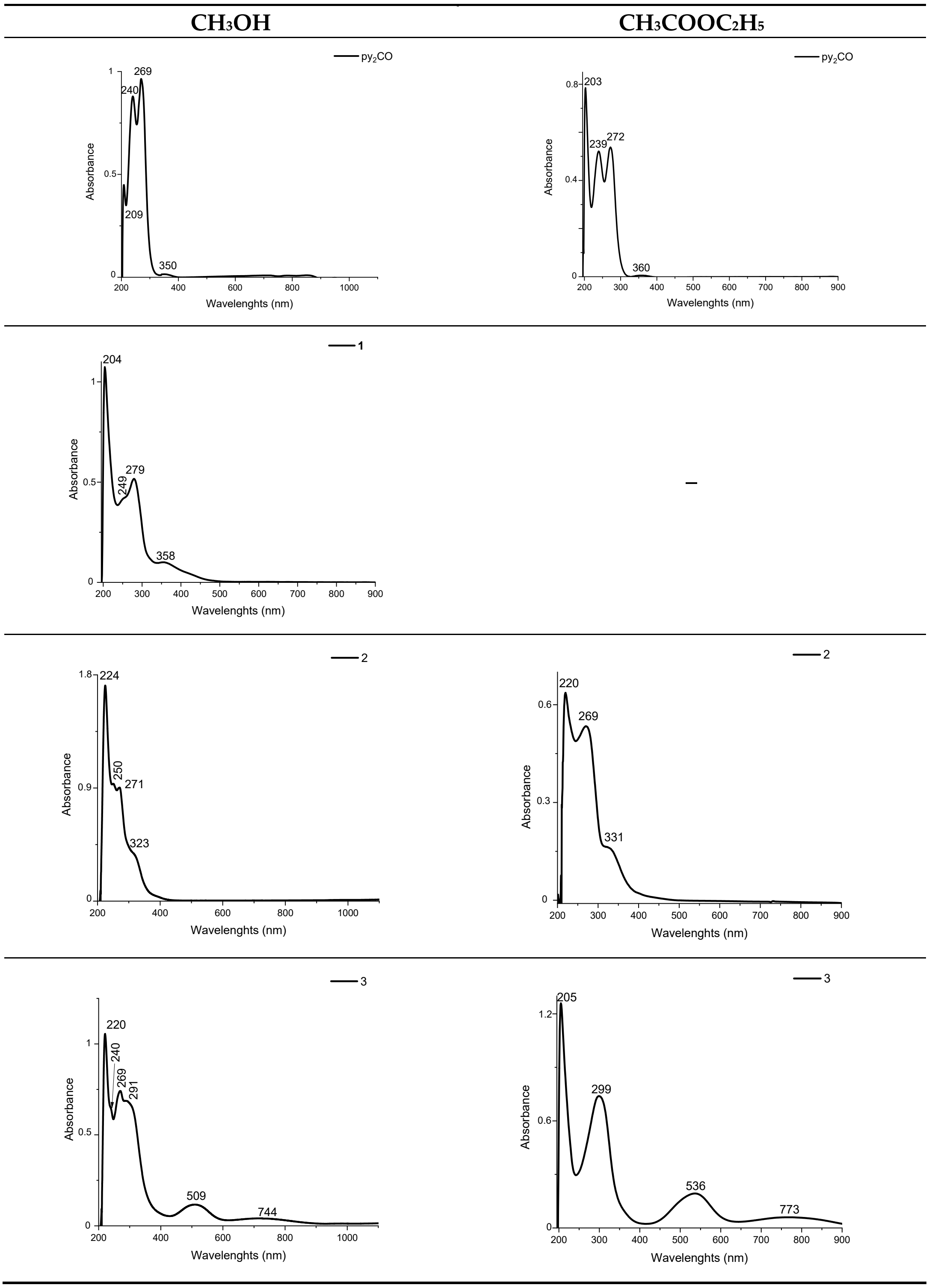

Figure 7. UV-Vis spectra of free ligand and analysed complexes in methanol and ethyl acetate.

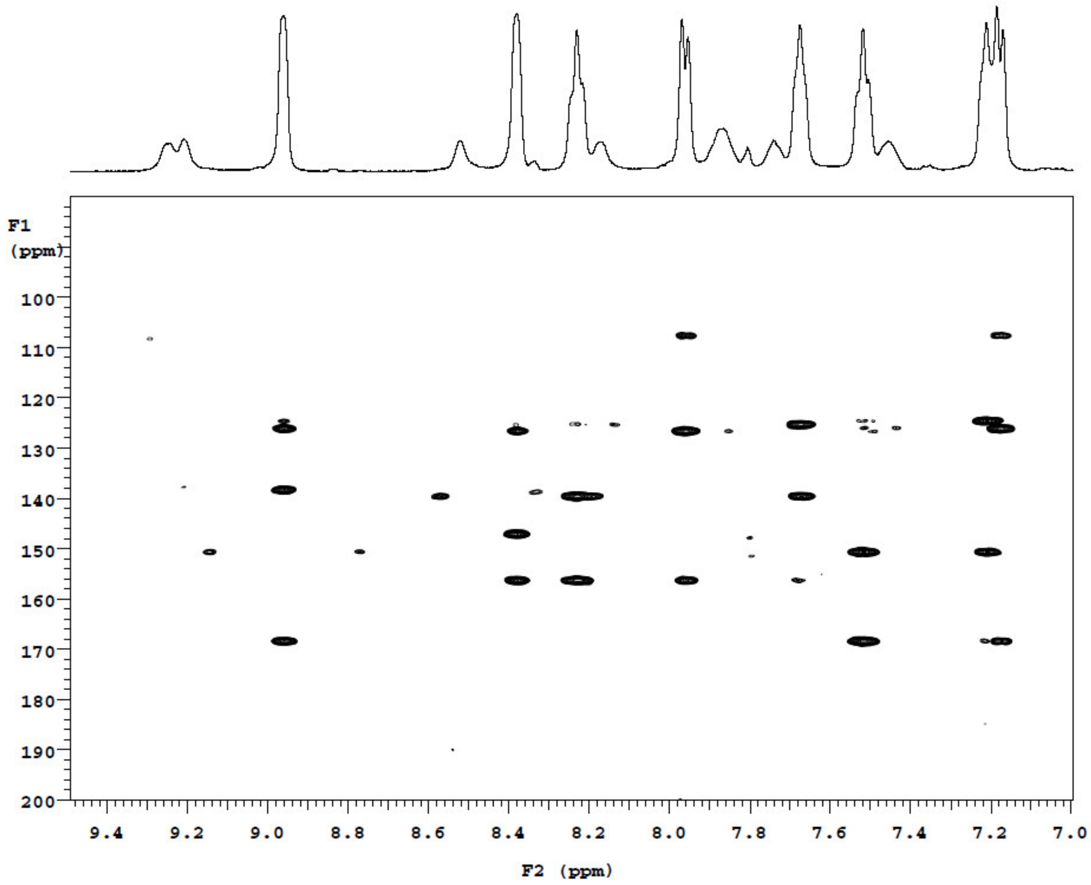

Figure 8. ^{1}H-^{13}C HMBC spectrum of **3** in D_2O at 278 K.

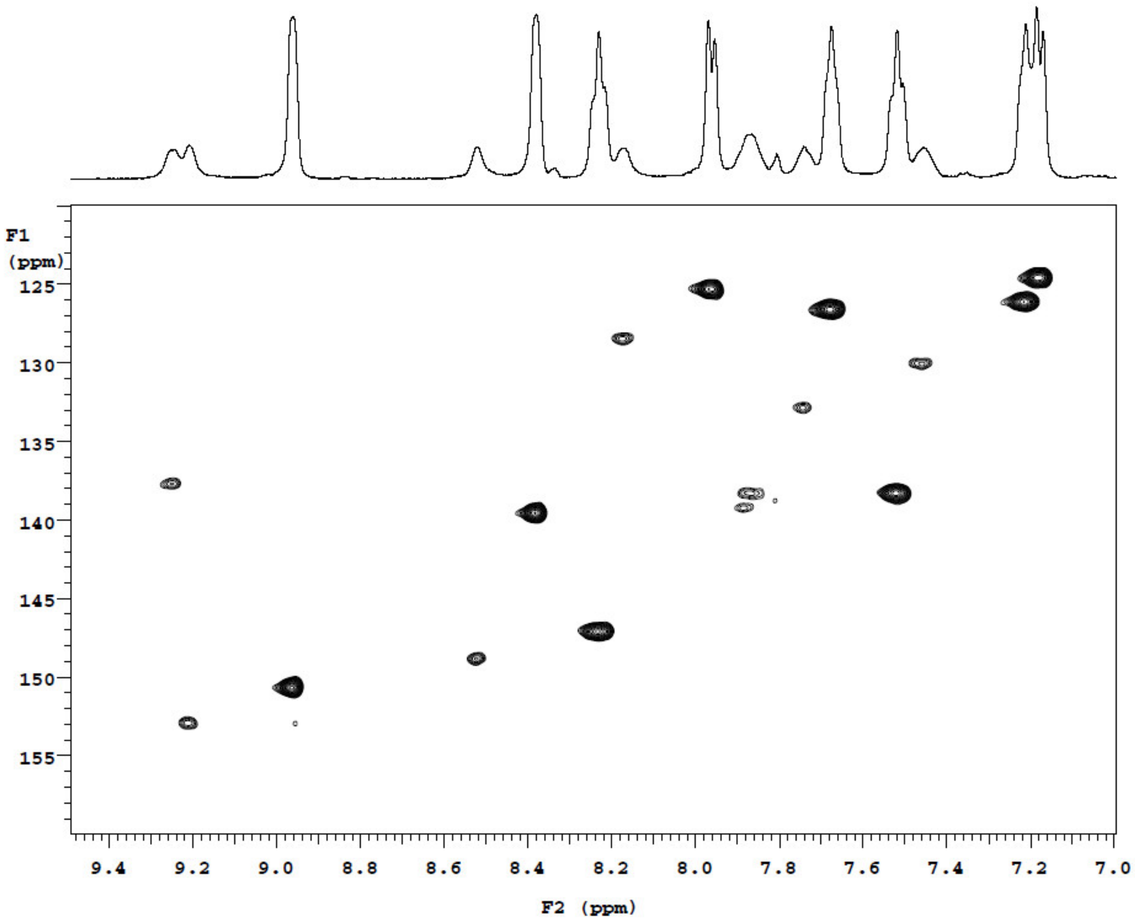

Figure 9. ^{1}H-^{13}C HSQC spectrum of **3** in D_2O at 278 K.

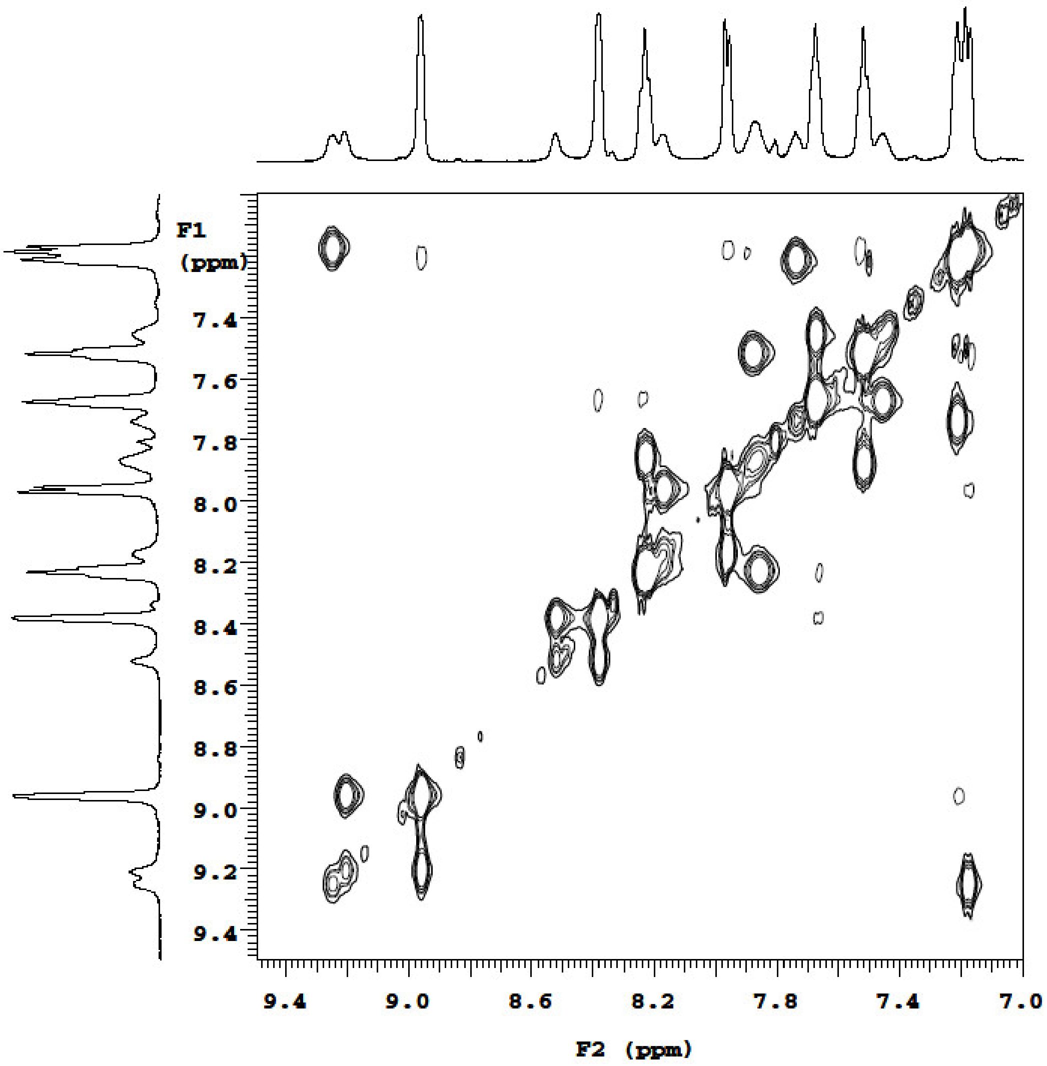

Figure 10. ^1H-^1H NOESY NMR spectrum of complex **3** in D_2O at low temperature (278 K) showing large exchange cross-peaks for being in equilibrium the main hydrolysis product.

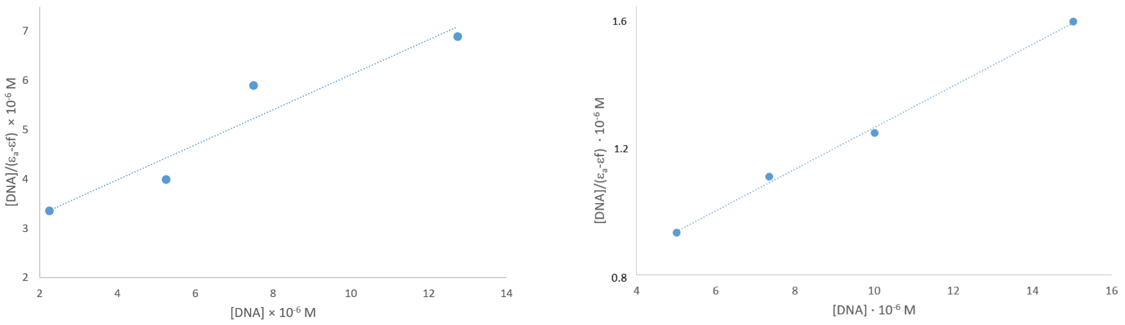

a) **b)**

Figure 11. Binding constant determination. Plots of [DNA]/(ε_a-ε_f) versus [DNA] a) **1** and b) **2**.

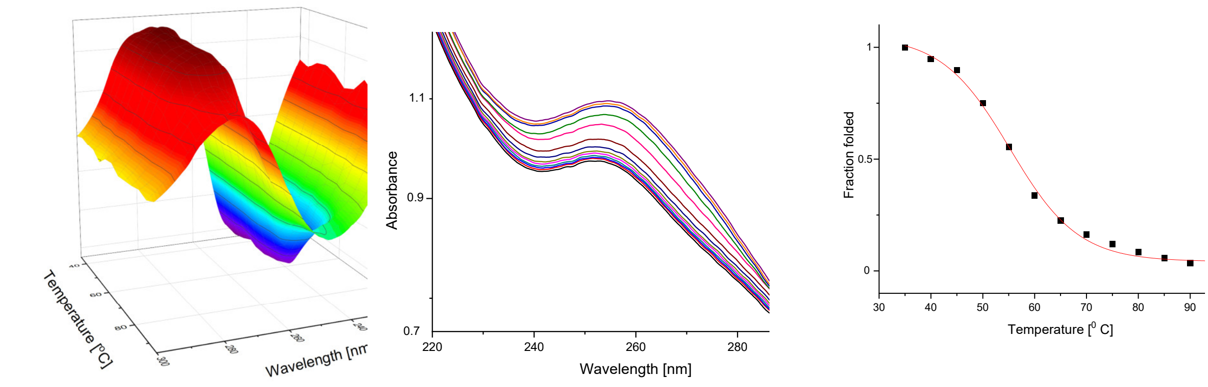

(**a**) (**b**) (**c**)

Figure S12. a) 3D CD melting of free CT-DNA (100 μM) in Tris-HCl/NaCl at pH 7.2, b) 2D absorbance spectra at 256 nm and c) fraction folded as function of temperature.

© 2020 by the authors. Submitted for possible open access publication under the terms and conditions of the Creative Commons Attribution (CC BY) license (http://creativecommons.org/licenses/by/4.0/).
